# Causal role of a neural system for separating and selecting multidimensional social cognitive information

**DOI:** 10.1016/j.neuron.2022.12.030

**Published:** 2023-04-05

**Authors:** Ali Mahmoodi, Hamed Nili, Caroline Harbison, Sorcha Hamilton, Nadescha Trudel, Dan Bang, Matthew F.S. Rushworth

**Affiliations:** 1 Wellcome Centre for Integrative Neuroimaging, Department of Experimental Psychology, University of Oxford, Oxford, UK; 2 Department of Excellence for Neural Information Processing, Center for Molecular Neurobiology (ZMNH), University Medical Center Hamburg-Eppendorf (UKE), Hamburg, Germany; 3 Max Planck UCL Centre for Computational Psychiatry and Ageing Research, London, UK; 4 Wellcome Centre for Human Neuroimaging, University College London, Oxford, UK; 5 Department of Experimental Psychology, University of Oxford, Oxford, UK; 6 Center of Functionally Integrative Neuroscience, Aarhus University, Aarhus, Denmark

**Keywords:** multidimensional social decision-making, fMRI, TMS, dorsomedial prefrontal cortex, anterior insula, non-invasive brain stimulation, cognitive map

## Abstract

People are multi-faceted, typically good at some things but bad at others, and a critical aspect of social judgement is the ability to focus on those traits relevant for the task at hand. However, it remains unknown how the brain supports such context-dependent social judgement. Here, we examine how people represent multidimensional individuals, and how the brain extracts relevant information and filters out irrelevant information when comparing individuals within a specific dimension. Using human fMRI, we identify distinct neural representations in dorsomedial prefrontal cortex (dmPFC) and anterior insula (AI) supporting separation and selection of information for context-dependent social judgement. Causal evaluation using non-invasive brain stimulation shows that AI disruption alters the impact of relevant information on social comparison, whereas dmPFC disruption only affects the impact of irrelevant information. This neural circuit is distinct from the one supporting integration across, as opposed to separation of, different features of a multidimensional cognitive space.

## Highlights

• AI and dmPFC activity patterns evolve during social decision-making
• AI-dmPFC interactions occur as relevant and irrelevant information is identified
• Disruption of dmPFC increases the impact of irrelevant information on choice
• Disruption of AI decreases the impact of relevant information on choice

## Teaser

Mahmoodi et al. identify distinct neural representations in dorsomedial prefrontal cortex (dmPFC) and anterior insula (AI) supporting separation and selection of information for context-dependent, multidimensional social judgement. The distributed pattern of activity complements those linked to integration of information. Disruption of dmPFC and AI produce distinct changes in social judgement.

## Introduction

People track how good they, and other people are, at performing different tasks. This process enables them to identify those with whom it would be best to cooperate as well as those with whom it would be best to compete or avoid competing.^1^^,^^2^^,^^3^ Because people are multi-faceted, it is also important to consider how the range of skills that each individual possesses might be exploited to compose the most effective multi-person teams.^4^ For example, when people consider the abilities of different potential team members on more than one dimension, they are able to identify the best combination of qualities or skills for the team.^4^ Just as in non-social situations,^5^^,^^6^^,^^7^^,^^8^^,^^9^^,^^10^ when combining multidimensional social information, people employ a “grid-like” cognitive map^4^—an abstract version of the maps used to represent the spatial environment that depend on the medial temporal lobe and medial prefrontal cortex.^11^

However, although we may frequently need to combine information across several dimensions of a multi-faceted individual, it is just as often the case that we must focus on one particular dimension. For example, imagine a prime minister who is aiming at filling different cabinet positions. When considering candidates for the secretary for education, the prime minister should focus on the candidates’ knowledge of education and not health or foreign policy. In other words, choosing the right individual in each context entails selecting the most relevant trait required for the job and, equally importantly, disregarding all the currently irrelevant information. The latter information might, however, become relevant when considering some of the same candidates for a different position. Similar considerations apply when we weigh up from whom it would be best to seek help in more everyday situations.

Here, we develop an experimental framework for studying how the human brain represents information about multi-faceted individuals in a flexible context-dependent manner, and how it selects and extracts relevant information and filters out irrelevant information. Participants first learned about seven characters’ abilities in each of three trait domains (contexts). For example, one character might have high mathematical ability but low memory and planning abilities. Participants then performed a two-alternative forced-choice task in which they compared pairs of characters within each trait domain—analogous to the prime minister seeking to select the best candidate for a particular position.

We show that although the ability to focus on specific social information exists, it is also limited: information in irrelevant social dimensions influences decision-making and especially so when there is little to distinguish two individuals in the relevant trait domain. To return to the prime minister analogy, when there is little to distinguish the candidates in terms of their knowledge of education, the prime minister might be swayed by differences in their knowledge of health or foreign policy. Using fMRI and continuous theta-burst stimulation in separate experiments, we identify distinct neural representations in dorsomedial prefrontal cortex (dmPFC) and anterior insula (AI) that underpin the selection and integration of social information, and we then determine their causal role in behavior by examining the impact of their disruption. Our results show that the neural circuit that underpins the ability to focus on specific social dimensions only partly overlaps with the circuit employed when combing information across social dimensions.^4^^,^^12^

## Results

### Experimental paradigm

**Figure 1:**
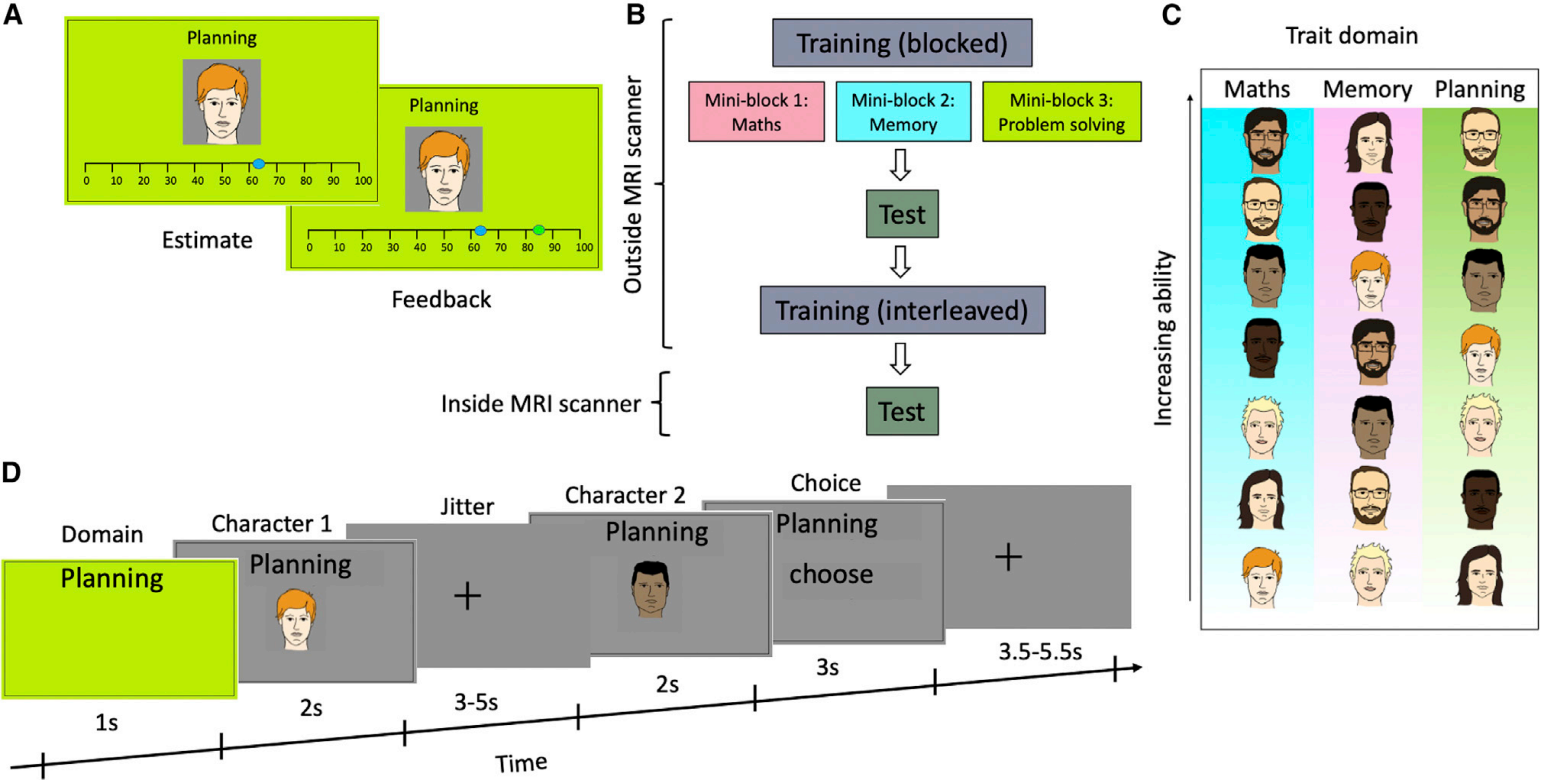
Experimental design The pre-scan session involved training and test blocks. (A–C) In training blocks, participants learned about the ability of the seven characters separately for each trait domain in the first training block and then in an interleaved manner in subsequent training blocks. (D) In test blocks, participants were on each trial first informed about the current trait domain and then presented with two characters sequentially. Participants had to choose the character with the higher ability in the specified trait domain. In the fMRI session, participants performed four test blocks (scan runs) and made all pairwise comparisons for each trait domain in each of these blocks.

**Figure 2:**
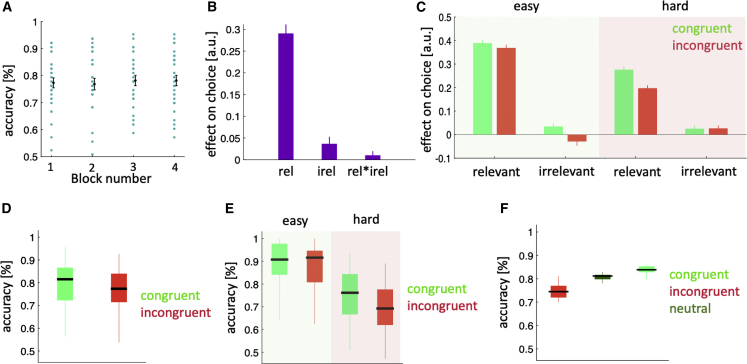
Behavioral results (A) Mean accuracy in each block. (B) Betas from a regression model in which we predicted participants’ choices (1: first character; 0: second character) using the difference between the first and second character in the relevant trait (rel), their average difference across the irrelevant traits (irel), block number and all possible interactions between these terms. (C) Regression betas from the same model as in (B) but applied separately for the four possible combinations of congruency and difficulty. (D) Mean accuracy separated by congruency. (E) Mean accuracy separated by congruency and difficulty. (F) Mean accuracy on congruent, neutral, and incongruent trials. In (A) error bars represent standard error of the mean (SEM). In (B) and (C), error bars represent 95% confidence interval (CI). In the boxplots in (D)–(F), the black vertical band indicates the median and the whiskers extend to the most extreme data samples that lie within the intervals spanning 1.5 times the inter-quartile range.

Participants (n = 30; experiment 1) performed a multidimensional social comparison task, first in a pre-scan session and then while undergoing fMRI (Figure 1). In the pre-scan session, participants learned about seven characters’ abilities in three trait domains; these domains were mathematics, memory and planning, and the ability of a character in one domain was independent of their ability in another one. To make the three trait domains as concrete as possible, participants performed tasks representative of each domain as part of the task instructions (see familiarisation in STAR Methods). In the fMRI session, participants were on each trial asked to select which of the two characters had higher ability within a given trait domain. To maximize choice accuracy, participants should compare the characters along the relevant trait only and ignore the two irrelevant traits. Participants completed four blocks (scan runs) and made all possible pairwise comparisons within each trait domain in each block (63 trials per block). Participants received feedback about their accuracy within each trait domain at the end of a block. In support of this design, participants were on average correct on 77% of trials (Figure 2A), and their performance did not decrease across blocks (Kruskal-Wallis H test, $\chi^{2}$(3) = 0.11, p = 0.99).

### Behavioral results

#### Effect of relevant and irrelevant traits on choice

We first assessed whether participants based their social comparisons on the relevant trait domain only or whether there was an additional influence of the irrelevant trait domains. To this end, we ran a logistic mixed effects model in which we predicted trial-by-trial choices using the parametric differences between characters along the relevant trait (relevant value difference) and irrelevant traits (irrelevant value difference) as well as their interaction (see LMM1 [Linear models for behavioral data analysis] in STAR Methods). In all linear regression models, we assessed the statistical significance of model parameters by F-statistics (see STAR Methods). In all other cases, we used Wilcoxon signed-rank test for hypothesis testing and applied Holm-Bonferroni correction (HBC) for multiple comparison, where applicable. As expected, the relevant trait had a positive effect: the larger the difference between the characters in the relevant trait domain, the more likely participants were to select the character with higher ability within this domain (Figure 2B; β ± 95% CI = 0.29 ± 0.03, F(1,29) = 371, p < 0.001, HBC). Notably, the irrelevant traits (defined as the average across irrelevant traits) also had a positive effect, with participants more likely to select the character of higher ability within the irrelevant trait domains (Figure 2B; β ± 95% CI = 0.03 ± 0.02, F(1,29) = 9, p < 0.01, HBC). Finally, there was a marginally significant positive interaction between relevant and irrelevant traits, indicating that the impact of the relevant value difference depended on the irrelevant value difference and vice versa (Figure 2B; β ± 95% CI = 0.01 ± 0.01, F(1,208) = 3.67, p = 0.05, HBC). We highlight that the differences between characters in relevant and irrelevant traits were uncorrelated, both by design and empirically (Figure S1). We also tested two alternative models, but our logistic model that we explained above provided a better fit (Figure S1).

We next sought to establish how the irrelevant trait domains interacted with the relevant trait domain during social comparison. One possibility is that the impact of the irrelevant traits depended on whether the sign of the difference between characters along the irrelevant traits was congruent with the sign of their difference along the relevant trait. Another, but not mutually exclusive, possibility is that the impact of the irrelevant traits depended on whether a given choice was easy or hard—with trials in which the absolute standardized difference between characters along the relevant trait was above 1 classified as easy and all other trials classified as hard.

To assess how the impact of the irrelevant traits varied across these task conditions, we re-ran our logistic mixed effects model separately for the four combinations of congruency and difficulty (congruent easy, congruent difficult, incongruent easy, and incongruent difficult). Consistent with the earlier results, the relevant trait had a positive impact on choice for all four trial types (Table 1). By contrast, the effect of irrelevant traits depended on the trial type (Table 1). On both types of congruent trials, the irrelevant traits had a positive impact on choice, further increasing the likelihood that the character of higher ability in the relevant trait domain was chosen. On incongruent easy trials, the irrelevant traits had a negative impact on choice, indicating that participants recognized the incongruency between the trait dimensions and actively decided against the character favored by the irrelevant traits (Figure 2C). Finally, on incongruent difficult trials, the irrelevant traits had a positive impact on choice (Figure 2C). In other words, when there was little difference between characters along the relevant trait, participants were more likely to base their choice on the difference along the irrelevant traits.

**Table 1:** Effects of the relevant and irrelevant information on choice

Analysis	Condition	β ± CI	F-statistics	p value
Effects of relevant information on choice	congruent easy	0.27 ± 0.01	F(1,1228) = 2,012	<0.001
	congruent-hard	0.47 ± 0.03	F(1,2392) = 1,057	<0.001
	incongruent easy	0.24 ± 0.01	F(1,1404) = 1,692	<0.001
	incongruent-hard	0.33 ± 0.03	F(1,2536) = 490	<0.001
Effects of irrelevant information on choice	congruent easy	0.06 ± 0.03	F(1,1228) = 13	<0.001
	congruent-hard	0.04 ± 0.02	F(1,2392) = 11	<0.001
	incongruent easy	−0.04 ± 0.03	F(1,1404) = 6	0.01
	incongruent-hard	0.03 ± 0.02	F(1,2536) = 8	0.003

Intuitively, the observed influence of irrelevant traits on incongruent difficult trials should impair participants’ performance. Indeed, participants’ accuracy was lower on incongruent than congruent trials (Figure 2D; W = 334, p = 0.03), and their accuracy was lower on incongruent difficult trials than congruent difficult trials (Figure 2E; W = 361, p < 0.01). These differences may be driven by disruption from the irrelevant traits on incongruent trials and/or facilitation from the irrelevant traits on congruent trials. To assess these accounts, we compared incongruent and congruent trials where the characters differed substantially along the irrelevant traits with another subset of trials where this difference was small and where there should be no disruption and/or facilitation (neutral trials). Specifically, incongruent and congruent trials only included trials where the absolute standardized difference along the irrelevant traits was above 0.5, whereas the remaining trials were labeled as neutral. This analysis indicated that both disruption and facilitation were at play (Figure 2F): participants’ accuracy was lower on incongruent than neutral trials (disruption W = 444, p < 0.001, HBC) and higher on congruent than neutral trials (facilitation W = 464, p < 0.001, HBC).

### Neuroimaging results

#### Distinct representations of relevant and irrelevant information in dmPFC and AI

We next turned to the fMRI data acquired during task behavior to understand how the brain represents relevant and irrelevant information and how these representations may interact during choice. To this end, we first estimated a whole-brain general linear model (GLM1) where the BOLD response to the first character was modulated by the character’s relevant and irrelevant traits and where the BOLD response to the second character was modulated by the difference between the chosen and unchosen characters along the relevant trait (relevant value difference) and irrelevant traits (irrelevant value difference). All clusters surviving whole-brain correction are shown in Tables S1 and S2.

**Figure 3:**
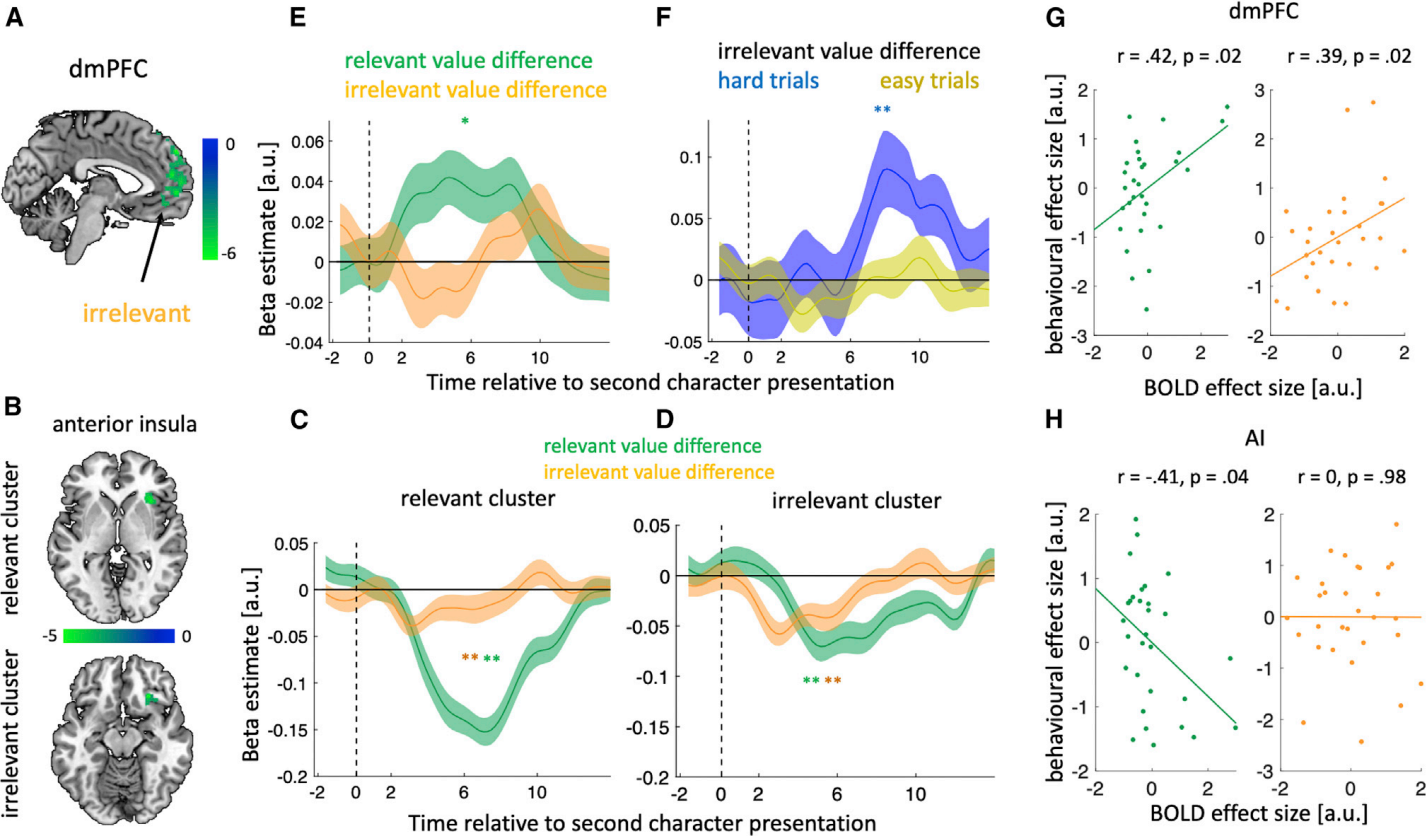
Distinct representations of relevant and irrelevant information in dmPFC and AI (A and B) Whole-brain analysis shows that (A), dmPFC activity at the presentation of the first character was negatively modulated by the character’s irrelevant traits (B), AI encoded the difference between the chosen and the unchosen character along the relevant trait (top left, relevant cluster) and irrelevant traits (bottom left, irrelevant cluster). (C and D) However, time course analyses showed that both relevant and irrelevant information were encoded in both relevant (C) and irrelevant (D) clusters. (E) dmPFC activity time courses encoded the difference between characters along the relevant trait (relevant value difference) but not along the irrelevant traits (irrelevant value difference) at the time of choice. (F) However, dmPFC activity time courses did encode the irrelevant value difference when the relevant value difference was small (hard trials) but not when it was large (easy trials). (G) Correlation between the effect of relevant information and (right) irrelevant information on the dmPFC BOLD response versus choice. (H) Analogous to (G) for AI. In (G) and (H), each dot represents a participant. In (A) and (B), we show clusters which are significant at p < 0.05, FWE-corrected for multiple comparisons; the cluster-defining threshold is p < 0.001, uncorrected. In (C)–(F), error bars represent standard error of the mean (SEM). ^∗^ p < 0.05, ^∗∗^ p < 0.01. p values of time course analyses were obtained using a leave-one-out procedure (see STAR Methods) and were corrected for multiple comparisons using Holm-Bonferroni correction (HBC), where applicable.

We first sought to unpack how irrelevant information enters the choice process; the behavioral results suggested that although the brain can filter out irrelevant information, it does not fully eliminate it. At the presentation of the first character, the whole-brain analysis identified a cluster in dmPFC that was negatively modulated by the character’s irrelevant traits: the higher the ability of the character along the irrelevant trait domains, the lower the dmPFC activity (Figure 3A). To confirm that dmPFC selectively tracked irrelevant information, we extracted dmPFC activity time courses and again predicted neural activity including both irrelevant and relevant traits. This analysis showed the expected effect of irrelevant traits but no effect of the relevant trait (Figure S2; W = 208, p = 0.6). One interpretation of the negative relationship between dmPFC activity and irrelevant traits is that dmPFC is involved in the suppression of irrelevant information—an interpretation which we return to in the non-invasive brain stimulation experiment below.

At second character presentation, the whole-brain analysis identified two clusters in AI: one cluster encoded the relevant value difference (relevant cluster; Figure 3B, top), whereas the other encoded the irrelevant value difference (irrelevant cluster; Figure 3B, bottom). However, time course analyses showed that both clusters tracked the relevant and the irrelevant value difference, albeit at slightly different points in time (Figures 3C and 3D; relevant cluster: relevant W = 1, p < 0.001, irrelevant W = 98, p < 0.01, irrelevant cluster: relevant W = 59, p < 0.001, irrelevant W = 51, p < 0.001). This result indicates that the whole-brain difference reflected the degree to which the clusters fitted the canonical BOLD response function used in our whole-brain analysis rather than a difference in computational function, and we therefore used the relevant cluster as our AI ROI for subsequent analysis. Notably, the relevant and irrelevant value differences both had a negative effect on AI activity. In Figure S3, we show that the negative encoding of the relevant value difference by AI can be decomposed into AI encoding the value of the chosen character with a negative sign and the value of the unchosen character with a positive sign, and we discuss how such a response pattern is indicative of a comparison process. Finally, we asked whether AI encodes relevant and/or irrelevant information at first character presentation. We found that AI tracked the relevant but not the irrelevant traits of the first character (Figure S2).

Given the involvement of dmPFC at first character presentation, we asked whether this area also carried value difference signals at second character presentation. Time course analyses showed that dmPFC activity was modulated by the relevant value difference but not by the irrelevant value difference (Figure 3E; relevant, W = 322, p = 0.03; irrelevant, W = 231, p = 0.31). However, the effect of irrelevant information might be different in hard compared with easy trials. In the hard trials, there is very little information to distinguish the characters based on their relevant traits which may pave the way for the irrelevant traits to enter the choice process. Indeed, when separating trials by difficulty, we found that dmPFC tracked the irrelevant value difference on hard but not on easy trials (Figure 3F; difficult, W = 364, p < 0.01; easy, W = 97, p = 0.99). We cannot quantify the impact of the relevant value difference separately for hard and easy trials as this categorization is itself based on the relevant value difference.

In summary, dmPFC and AI encoded information in distinct but complementary ways. On one hand, dmPFC tracked the irrelevant trait at the time of the first character presentation and the difference between characters in the relevant trait domain at second character presentation. Notably, dmPFC also encoded the difference between the characters in the irrelevant trait domain when there was little to distinguish them in the currently relevant domain (hard trials). On the other hand, AI encoded the relevant trait at the time of first character presentation and the relevant and irrelevant value difference at second character presentation. These response profiles differ in two key aspects: (1) at the time of first character presentation, AI encodes relevant information, whereas dmPFC encodes irrelevant information and (2) once the second character has been presented, the encoding of irrelevant information by AI is trial-independent, whereas the encoding by dmPFC is trial-dependent as it is only present in hard trials. These results indicate that AI supports the amplification of relevant information, whereas dmPFC supports the separation of the irrelevant and relevant information and the suppression of irrelevant information.

Finally, we asked whether the degree to which these regions tracked relevant and irrelevant traits predicted the impact of these traits on behavior. To address this question, we applied separate regression models for each participant which quantified the parametric impact of relevant and irrelevant traits (as well as their interaction) on (1) dmPFC activity, (2) AI activity, and (3) choice. We then examined the correlation between (1–2) the returned regression coefficients quantifying the parametric impact of relevant and irrelevant traits on dmPFC or AI activity with (3) the regression coefficients obtained for choice. In dmPFC, for both the relevant and irrelevant traits, larger neural responses predicted larger behavioral effects (Figure 3G; relevant, Pearson r = 0.42, p = 0.02; irrelevant, Pearson r = 0.39, p = 0.02, HBC). In AI, a smaller neural response predicted a larger behavioral effect for the relevant trait, whereas there was no correlation between AI activity and choice when considering the irrelevant trait (Figure 3H; relevant, Pearson r = −0.41, p = 0.04; irrelevant, Pearson r = 0, p = 0.98; HBC). These results are consistent with the interpretation that AI supports the amplification of relevant information, whereas dmPFC supports the separation of irrelevant and relevant information and, in particular, the suppression of irrelevant information.

#### Rich task representations in dmPFC and AI

To fully characterize the neural representations carried by dmPFC and AI, we next applied representational similarity analysis (RSA), a multivariate method that considers the pattern of activity rather than the mean activity within a neural area.^13^^,^^14^

**Figure 4:**
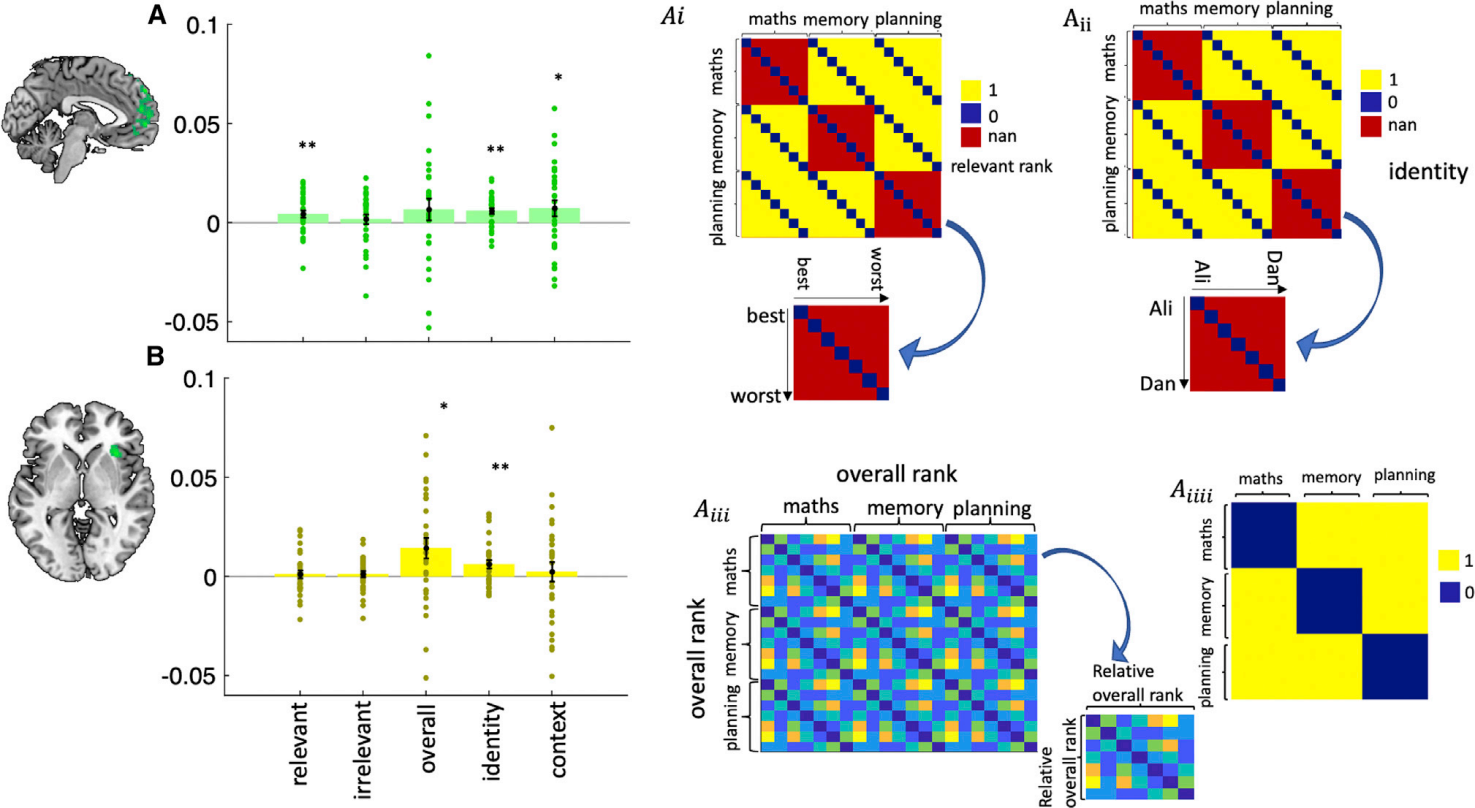
Rich task representations in dmPFC and AI identified by RSA (A) Bar plot shows correlations (*Kendall’s*$\tau_{A}$) between neural representational dissimilarity matrices (RDMs) obtained from dmPFC and five different behavioral RDMs (illustrations from top to bottom: relevant rank, character identity, overall rank and context). Apart from replacing relevant ranks with irrelevant ranks, there was no other difference between relevant and irrelevant RDMs. Each dot is a subject. (B) Same as in (A) but for AI. In (A) and (B), p values were corrected for multiple comparison using Holm-Bonferroni correction (HBC). Error bars represent standard error of the mean (SEM). ^∗^ p < 0.05, ^∗∗^ p < 0.01.

We first asked whether multivariate dmPFC activity carried information about the relevant trait, by testing whether the activity patterns for characters of comparable relevant trait rank were more similar than for characters of different relative trait rank. Although univariate dmPFC activity was not modulated by the relevant trait but by the irrelevant traits (Figure 3A), relevant rank information was encoded by multivariate dmPFC activity (Figure 4A; W = 329, p < 0.01, HBC), whereas irrelevant rank information was not (Figure 4A; W = 290, p = 0.24, HBC). We next asked whether dmPFC carried an overall impression of the characters, defined as their overall rank across all three trait domains. However, we found no evidence for such representation (Figure 4A; W = 301, p = 0.15, HBC). Finally, we asked whether dmPFC carried more general information about the task, such as the identity of the trait domains (contexts) or the characters. Indeed, multivariate dmPFC activity discriminated between contexts (Figure 4A; W = 309, p = 0.05, HBC) as well as characters regardless of the current context (Figure 4A; W = 396, p < 0.001, HBC).

We next applied the same analyses to AI. Multivariate AI activity did not carry information about the relative trait rank of characters within the current context (Figure 4B; W = 238, p = 0.9, HBC), their irrelevant trait rank (Figure 4B; W = 279, p = 0.34, HBC) or the current context itself (Figure 4B; W = 254, p = 0.65, HBC). However, multivariate AI activity carried information about the overall character rank across all three trait domains (Figure 4B; W = 357, p = 0.01, HBC) and character identity (Figure 4B; W = 361, p < 0.01, HBC). In summary, multivariate dmPFC and AI activity carried complementary task representations. Although dmPFC represented character identity regardless of the current context, it also represented the currently relevant trait domain and the relative trait rank of characters within the current context—task features that are critical for choice on any given trial. In contrast, AI represented character identity, regardless of the current context as well as overall character rank across all three trait domains—task features that are relevant for the task more generally but not for choice on any particular trial. In Figure S4, we show that dmPFC carries not only a categorical but also a parametric rank representation in the relevant trait domain. In other words, dmPFC not only discriminates between the different ranks but is also sensitive to the ordered relationship between ranks.

#### No 3D representation of social hierarchy space

The question of how the brain represents not only physical but also cognitive spaces, such as the social hierarchy space in our task, has attracted considerable attention in recent years.^6^ Previous studies have shown that medial temporal regions (hippocampus and entorhinal cortex: HC-EC system) and medial prefrontal areas carry a map-like representation of two-dimensional (2D) social hierarchy spaces in which individuals are organized according to their Euclidian distances in the 2D space.^4^^,^^12^^,^^15^ Given the interest in such cognitive maps and the unique opportunity afforded by our three-dimensional (3D) social hierarchy space, we applied a whole-brain RSA searchlight procedure in which we sought to identify neural areas that organized the characters according to their Euclidian distances in a 3D space. However, we also reasoned that the brain might use a different coding scheme in such a 3D space which is more complex than the 2D spaces used in previous social studies—just as the representation of 3D physical space in free flying bats is different from the representation of 2D physical space which has been much studied in rodents in 2D spatial arenas.^16^ We therefore also searched for neural areas that carried a compressed social hierarchy representation where the characters were organized according to their overall rank as in our ROI analysis.

Our whole-brain RSA searchlight procedure did not identify any regions that carried a 3D representation of social hierarchy space. However, in line with our ROI analysis, it identified a cluster in the border between AI and frontal operculum that encoded the overall rank of the characters (Figure S5). In addition to dmPFC and AI, we also directly tested for a 3D representation in the HC-EC system using anatomically defined masks. However, we did not find evidence for a 3D representation of social hierarchy space in any of our ROIs (Figure S6; EC, W = 248, p = 0.37; HC, W = 209, p = 0.68; AI, W = 239, p = 0.45, dmPFC, W = 187, p = 0.82). In Figure S6, we show that these null results are more likely to reflect a genuine lack of an effect than a lack of statistical power. For completeness, we examined whether the EC-HC system may have tracked the 3D difference (difference between the two characters across all three domains) between characters at the time of choice. Again, we found no evidence for such representation of social hierarchy space (Figure S6; EC, W = 225, p = 0.99; HC, W = 265, p = 0.5).

#### Functional connectivity between AI and dmPFC

Finally, we reasoned that the ultimate character selection should depend on an interaction between the social representations carried by dmPFC and AI. Notably, identifying the relevant information is more critical on incongruent than congruent trials; on incongruent trials, the relevant and irrelevant traits favor different choices, whereas on congruent trials, judging the characters based on overall rank, or even irrelevant traits, would lead to the same choice. For this reason, we would expect connectivity between dmPFC and AI to vary with congruency in addition to the relevant and irrelevant value differences. We therefore deployed a psychophysiological interaction analysis in which we predicted dmPFC activity using the interaction between AI activity and (1) the relevant value difference, (2) the irrelevant value difference and (3) the interaction between the relevant and the irrelevant value differences.

**Figure 5:**
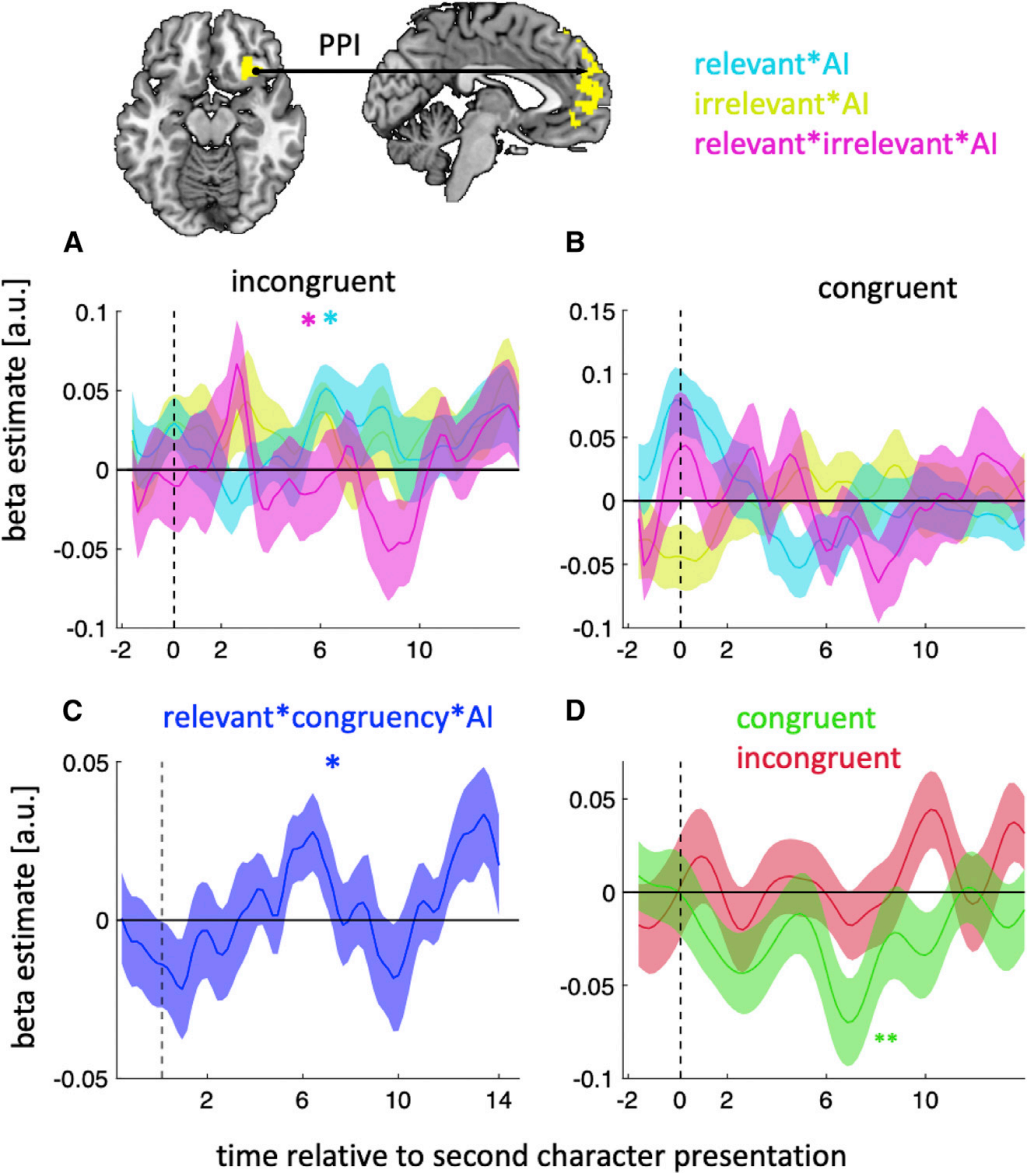
Functional connectivity between AI and dmPFC (A) AI-dmPFC connectivity increased with the difference between the chosen and unchosen characters in the relevant trait domain in incongruent trials. (B) This increase was not seen in congruent trials. (C) There was a significant difference in AI-dmPFC connectivity between congruent and incongruent trials as a function of the difference between the chosen and unchosen characters in the relevant trait domain. (D) The effect of irrelevant information in AI was only present in congruent not incongruent trials. (A–D) Error bars represent standard error of the mean (SEM). ^∗^ p < 0.05.

As predicted, in incongruent trials, AI-dmPFC connectivity increased with the relevant value difference (Figure 5A; W = 339, p = 0.04, HBC) as well as the interaction between the relevant and irrelevant value differences (blue in Figure 5A; W = 330, p = 0.04, HBC). In contrast, in congruent trials, AI-dmPFC connectivity did not vary with the relevant value difference (Figure 5B; W = 291, p = 0.22, HBC). Supporting the contrast between trial types, an analysis that combined both trial types showed that AI-dmPFC connectivity varied with an interaction between congruency and the relevant value difference (Figure 5C; W = 130, p = 0.03). The change in dmPFC-AI connectivity, which is specific to incongruent trials might suggest that AI and dmPFC eliminate the effect of irrelevant information in incongruent trials, where its presence can impair the comparison between characters. To test this possibility, we quantified the effect of irrelevant information on AI activity separately for congruent and incongruent trials. Intriguingly, we found no effect of irrelevant information on incongruent trials (Figure 5D; W = 349, p = 0.99, HBC), whereas its effect remained significant on congruent trials (W = 101, p < 0.01, HBC) and was significantly stronger than on incongruent trials (W = 397, p < 0.001). Finally, we note that the change in AI-dmPFC connectivity in incongruent trials (Figure 5A) is consistent with an account in which AI first contributes to the suppression of irrelevant information in dmPFC (pink peak around 2 s) and then the amplification of relevant information (blue peak around 8 s).

#### Causal manipulation of dmPFC and AI

**Figure 6:**
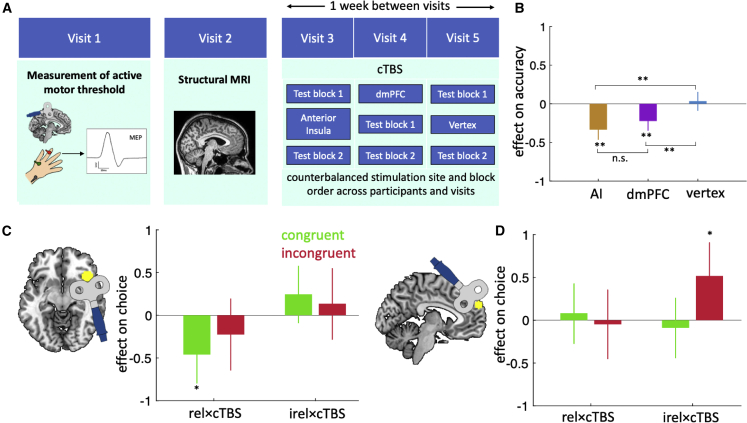
Causal manipulation of dmPFC and AI (A) cTBS procedure. Each participant attended five sessions: we (visit 1) determined their active motor threshold, (visit 2) obtained a structural MRI scan and (visits 3–5) applied cTBS to dmPFC, AI or vertex in one of the two test blocks in each session. Block order and session order were pseudo-randomized. (B) cTBS in AI and dmPFC, but not vertex, led to a decrease in performance. (C) AI cTBS caused a decrease in the effect of the relevant value difference on congruent trials. (D) dmPFC cTBS caused an increase in the effect of the irrelevant value difference on choice on incongruent trials. In (B)–(D), error bars represent 95% confidence interval (CI). ^∗^ p < 0.05, ^∗∗^ p < 0.001.

To establish causal roles of dmPFC and AI in the separation and selection of multidimensional social cognitive information, we ran a within-subject experiment (n = 14, experiment 2) where we applied continuous theta-burst stimulation (cTBS) directed toward AI, dmPFC, and a control area (vertex) during separate visits to the laboratory (Figure 6A). In each of these sessions, participants performed two test blocks; we applied stimulation either before the first block with a 40-min break before the second block or 40 min after the first test block and just before the second block. We note that the AI clusters identified by our whole-brain analysis spread laterally along the surface of the frontal operculum, which is accessible to transcranial magnetic stimulation (TMS),^17^ as opposed to along the insular surface per se, which may be inaccessible to TMS (Figure S7). Inevitably the frontal operculum area, which is closer to the TMS coil, is also affected by the TMS pulses together with the underlying AI cortex. Thus, in the following, when we report the effect of TMS targeted at AI, it is important to remember both that BOLD activity spread beyond AI into frontal operculum and that frontal operculum stimulation occurred simultaneously with AI stimulation.

We first considered the impact of cTBS on task performance. To this end, we ran a linear regression model in which we predicted choice accuracy using (1) the relevant and irrelevant value differences, (2) whether cTBS was applied before a block of trials, (3) block number, and (4) all possible interactions between these terms (see LMM2 [Linear models for cTBS experiment] in STAR Methods). We included block number to control for changes in performance due to the 40-min break between blocks. This analysis showed that cTBS over both AI and dmPFC led to a reduction in accuracy (Figure 6B; AI, β ± 95% CI = −0.33 ± 0.12, F(1,1764) = 27, p < 0.001; dmPFC, β ± 95% CI = −0.22 ± 0.12, F(1,1728) = 12.75, p < 0.001, HBC), whereas cTBS over vertex had no impact (Figure 6B; β ± 95% CI = 0.03 ± 0.11, F(1,1764) = 0.3, p = 0.58, HBC). To compare the areas against each other, we conducted a separate regression model which included all possible pairs of areas (three separate regression models for three pairs of areas: dmPFC vs. AI, AI vs. vertex, dmPFC vs. vertex). The results of these regression models showed that although the interaction between area and cTBS was not different for dmPFC versus AI (β ± 95% CI = −0.11 ± 0.17, F(1.3492) = 1.56, p = 0.21), this effect was different for AI versus vertex (β ± 95% CI = −0.36 ± 0.18, F(1.3528) = 17.3, p < 0.001) and for dmPFC versus vertex (β ± 95% CI = −0.31 ± 0.18, F(1,3492) = 12, p < 0.001). Overall, cTBS over our target areas (AI and dmPFC) impaired participants’ ability to choose the better of the two characters, but this impairment could not be attributed to any peripheral or side effect of cTBS because it was absent for the control site (vertex).

The performance impairment caused by AI and dmPFC cTBS could reflect a decreased effect of relevant information and/or an increased effect of irrelevant information. To tease apart these competing factors, we applied logistic mixed effects regression models to choice but now separately for each of the four combinations of difficulty and congruency. Given our earlier behavioral and neural results, we here focus on hard trials—where the effect of cTBS should be more apparent—and report on easy trials in Table S3.

Our analysis indicated that cTBS over AI and dmPFC had different effects on the impact of relevant and irrelevant information on choice. For AI, cTBS reduced the impact of *relevant* information on *congruent* trials (Figure 6C; β ± 95% CI = −0.46 ± 0.35, F(1,588) = 4.5, p = 0.03, HBC), but it did not alter the effect of relevant information on incongruent trials (β ± 95% CI = −0.27 ± 0.43, F(1,470) = 1.6, p = 0.4, HBC) or the effect of irrelevant information on either trial type (congruent, β ± 95% CI = 0.24 ± 0.34, F(1,588) = 1.85, p = 0.51; incongruent, β ± 95% CI = 0.16 ± 0.42, F(1,470) = 0.55, p = 0.45; HBC). In contrast, for dmPFC, cTBS increased the impact of *irrelevant* information on *incongruent* trials (Figure 6D; β ± 95% CI = 0.51 ± 0.4, F(1,495) = 6.5, p = 0.04, HBC), but it did not alter the effect of irrelevant information on congruent trials (β ± 95% CI = −0.08 ± 0.35, F(1,622) = 0.24, p = 0.62, HBC) or the effect of relevant information on either trial type (congruent, β ± 95% CI = 0.08 ± 0.35, F(1,622) = 0.2, p = 0.65; incongruent, β ± 95% CI = −0.04 ± 0.41, F(1,495) = 0.05, p = 0.81; HBC). Notably, a separate regression model, which combined congruent and incongruent trials, identified a three-way interaction between irrelevant information, dmPFC cTBS and congruency (β ± 95% CI = −0.59 ± 0.52, F(1,1117) = 4.82, p = 0.02; HBC). This result supports the interpretation that cTBS over dmPFC specifically increases the influence of irrelevant information on incongruent trials—where considering irrelevant information is more detrimental—and highlights a central role for dmPFC in insulating the choice process from irrelevant information. In addition, in Figure S8 and Tables S4, we report results from separate regression analyses of “cTBS” and “No cTBS” blocks. We note that the impact of dmPFC or AI cTBS on a participants’ choice accuracy did not correlate with their choice reaction time as measured in the No cTBS blocks (Pearson correlation; AI: r = 0.08, p = 0.76; dmPFC: r = −0.16, p = 0.56).

Finally, to directly compare the effects of cTBS over AI and dmPFC, we ran a linear regression model in which we included both areas and tested for three-way interactions between (1) relevant or irrelevant information, (2) cTBS, and (3) area. This analysis identified a significant three-way interaction for relevant information on congruent trials (β ± 95% CI = −0.53 ± 0.49, F(1,1258) = 4.63, p = 0.03; HBC), in line with the suggestion that the reduction in the impact of relevant information after AI cTBS was different from that found after dmPFC cTBS. However, the three-way interaction for irrelevant information on incongruent trial did not reach significance (β ± 95% CI = −0.4 ± 0.48, F(1,999) = 1.94, p = 0.16; HBC).

## Discussion

This study investigated how the brain represents social environments where individuals—as in the real world—vary along multiple dimensions. The nature of our neural representations might depend on the complexity of the social information, such as the number of dimensions, and the types of operation performed on these representations, for example, whether we must evaluate individuals along a single dimension or across several dimensions.

**Figure 7:**
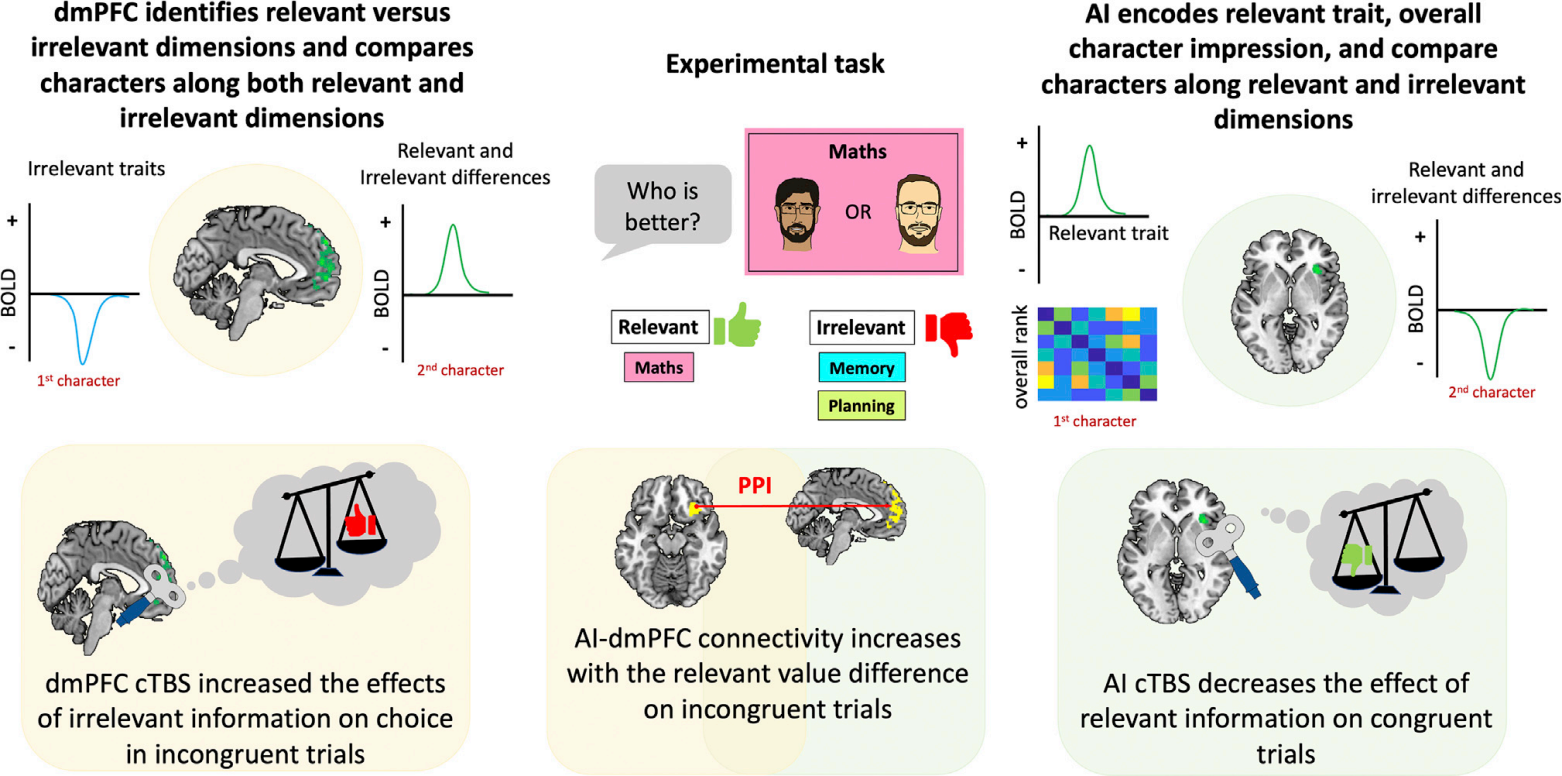
Summary dmPFC and AI play distinct roles in multidimensional social comparison.

We identified two regions, dmPFC and AI, that were central to the separation and selection of multidimensional social cognitive information (Figure 7). Variables that were relevant and irrelevant for choice were reflected in dmPFC activity. In particular, dmPFC activity decreased with the magnitude of the irrelevant traits at the time of first character presentation, but then increased with the difference between the characters in irrelevant traits on hard trials at second character presentation. Disruption of dmPFC reduced the ability to select the character favored by the relevant trait. Critically, this reduction was not due to a noisier comparison process but instead driven by an increased impact of irrelevant traits in hard incongruent trials. This suggests dmPFC mediates context-dependent separation of relevant and irrelevant social information.

The response profile of AI was notably different from that of dmPFC. At the time of first character presentation, multivariate AI activity encoded variables that were not relevant for choice, including character identity and overall character rank. Once the second character had been presented, univariate AI activity encoded the difference between the characters in both the relevant and irrelevant traits, although the encoding of the irrelevant difference was only present on congruent trials where it should increase, rather than decrease, the probability of selecting the right character. In support of an interpretation that AI supports the amplification of currently relevant information, causal disruption of AI using cTBS also reduced the ability to select the character favored by the relevant trait, but, unlike dmPFC, this reduction was driven by a decreased impact of the relevant trait, and specifically when the relevant and irrelevant traits favored the same character. We note that, when cTBS was applied to AI, it would also have affected the adjacent frontal operculum, which is cloer to the coil than AI.

Taken together, our results suggest that multidimensional social comparisons are supported by a neural network in which dmPFC underpins the context-dependent separation of relevant and irrelevant information and in which AI underpins the selection of the relevant information upon which the current choice is based. This account is further supported by the pattern of coupling between dmPFC and AI: univariate AI-dmPFC functional connectivity increased on incongruent trials—where there is the greatest need for separating relevant and irrelevant information—and specifically as a function of the currently relevant difference between the characters—a variable whose amplification has a greater benefit on incongruent trials.

The dmPFC is considered a key node in the brain’s so-called theory-of-mind network that underpins social cognition.^18^ A set of recent studies showed that dmPFC supports the representation of others’ ability as distinct from one’s own^2^ and that cTBS over dmPFC disrupts this self-other distinction.^3^ Our study shows that dmPFC is critical for distinguishing not only between self and others but also between different other individuals and that this social representation flexibly adapts to the current context. We highlight that although dmPFC is not uniquely concerned with social cognition, neural encoding of non-social information is usually strongest more ventrally within medial prefrontal cortex, whereas social information is most prominent in the region identified here.^19^ Neural representations within this region also typically carry more detailed information about social compared with non-social stimuli.^20^^,^^21^

The hypothesis that the brain uses map-like representations—cognitive maps—to organize spatial and non-spatial knowledge has received increasing empirical support,^6^ including in the social domain.^4^^,^^12^^,^^15^ However, we found no evidence for such a representation, including in ROI-based analyses of the HC-EC system identified by previous studies. There are at least two possible, but not mutually exclusive, explanations for this discrepancy: the brain may use other coding schemes (1) when tasked with a 3D rather than the 2D spaces used in earlier studies and/or (2) when separation of dimensions, as opposed to integration across dimensions, is required. Indeed, although not carrying a 3D representation as such, one possibility is that AI instead encoded a weighted sum across dimensions—as supported by AI encoding the overall rank of the first character—and that the weighting of each dimension then changed according to the requirements of the current decision—as supported by AI tracking mainly the relevant difference between the two characters at the time of choice.

The dmPFC-AI network described here is distinct from the network identified by research on non-social context-dependent multidimensional choice. Regions activated by non-social tasks include ventromedial PFC, which supports the construction of stimulus value from both single and multiple stimulus attributes,^7^^,^^22^^,^^23^^,^^24^^,^^25^ and dorsolateral PFC, which tracks the need for integrating stimulus attributes into an overall stimulus value^25^ and for filtering out currently irrelevant stimulus attributes.^26^ However, in contrast to our study, these tasks have not required participants to flexibly switch between different stimulus attributes^25^ or used a stimulus space where a currently irrelevant attribute may reverse judgements about the currently relevant attribute.^26^ As is true for most social neuroscience studies, it is possible that one could devise a non-social task analogous to ours and find that it recruits similar neural mechanisms. However, such a result would not alter the importance of the current results for understanding the neural basis of social behavior.

Although our study brings the multidimensional nature of social judgement under experimental control, it involves simplifications that should be addressed in future work. First, participants were told which trait domain to consider for social comparison. However, in many everyday situations, the currently relevant context must be inferred from ambiguous cues. Second, participants were pre-trained on individual traits and the behavioral manifestation of these traits was static. Although skill-related behaviors may be stable, personality-related behaviors can be volatile. It remains to be seen how the brain solves the problem of multidimensional social judgement when faced with context uncertainty and/or trait domains associated with high levels of behavioral variance. Third, the trait domains in our study were, by design, independent, but there is evidence that individual traits may be correlated across domains in the real world.^27^^,^^28^ Such a social prior is unlikely to account for the full set of fMRI and cTBS results, but it may be one reason why participants did not fully filter out currently irrelevant traits. Finally, the ability to separate out other people’s traits is beneficial in our task, but this may not always be the case. In the real world, non-holistic social inference may be associated with negative social interaction styles and dark-triad personality traits such as Machiavellianism.^29^

## STAR★Methods

### Key resources table

**Table:** 

REAGENT or RESOURCE	SOURCE	IDENTIFIER
**Deposited data**		

Raw and analysed data	This paper	https://doi.org/10.6084/m9.figshare.21510831.v1

**Software and algorithms**		

MATLAB R2020b	MathWorks	RRID:SCR_001622 https://www.mathworks.com/
SPM12	Wellcome Trust Centre for Neuroimaging	RRID:SCR_007037 https://www.fil.ion.ucl.ac.uk/
Brainsight	Rogue Research	https://www.rogue-research.com/
Spike2 Software	Cambridge Electronic Design Limited	https://ced.co.uk

**Others**		

Magstim Rapid2 stimulator (TMS)	Magstim	https://www.magstim.com/
D440-2 Isolated EMG amplifier	Digitimer	https://www.digitimer.com/
Hum Bug 50/60 Hz Noise Eliminator	Quest Scientific	https://www.digitimer.com/
CED power1401	Cambridge Electronic Design Limited	https://ced.co.uk

### Resource availability

#### Lead contact

Further information and requests for resources and reagents should be directed to and will be fulfilled by the lead contact, Ali Mahmoodi (ali.mahmoodi1367@gmail.com).

#### Materials availability

Materials are available from the lead contact upon reasonable request.

### Experimental model and subject details

#### Participants

Thirty participants (mean age ± standard deviation = 24 ± 4, 13 female) took part in the fMRI study (Experiment 1). 20 participants were recruited into the cTBS study (Experiment 2), six of whom were excluded from data analysis due to terminating the experiment following the pre-experimental procedure (n = 4), an active motor threshold above 50% of the transcranial magnetic stimulation machine’s total output (n = 1) or performance in the experimental task at chance level (n = 1)). The final sample comprised 14 participants (mean age ± standard deviation = 23±5, range 18-35, 8 female). All participants gave their informed consent before each session and were reimbursed for their participation. The study was approved by the central Research Ethics Committee at the University of Oxford (R60547/RE001).

#### Experimental Procedure

We conducted two experiments. In Experiment 1, we used fMRI to assess the neural correlates underpinning the selection and integration of social information. In Experiment 2, we used continuous theta-burst stimulation (cTBS) to disrupt activity in the dmPFC and AI to determine their causal role in behavior. The two experiments used a different sample of participants.

##### Experiment 1 (fMRI)

Participants completed one fMRI session. The session lasted approximately two hours. The behavioral task consisted of a training and a test part. Participants undertook 2 training blocks and 1 test block, before completing 4 test blocks within a 3T MRI scanner. Each block comprised 63 trials, and the fMRI scan lasted for approximately 60 minutes. Participants were told that their final payment would be computed based on their accuracy in the task. Every participant was paid £15/hour. In addition, all participants were paid up to £20 bonus in each session which was calculated based on their performance (£0 for 50% accuracy and £20 for 100% accuracy).

##### Experiment 2 (cTBS)

The second experiment included five sessions. In Session 1 (30 mins), participants attended a short “taster” session during which we measured their active motor threshold for the left motor cortex "hotspot", i.e., the scalp location where single-pulse transcranial magnetic stimulation evoked the largest motor-evoked potential (MEP) amplitude in the right first dorsal interosseous (FDI).^30^ The active motor threshold was defined as the maximum stimulation intensity needed to produce an MEP in the FDI in at least 50% of pulses while participants exerted constant pressure (around 10% of their maximum force) between their index finger and thumb. MEPs were recorded using bipolar surface Ag-AgCl electrodes.

Additionally, we applied 10 seconds of continuous theta-burst stimulation (cTBS) approximately at the dmPFC at a low intensity to serve as an introduction to the stimulation that would be experienced in the three experimental sessions. In Session 2 (30 mins), participants underwent a whole-brain anatomical 3T MRI scan that would later be used to guide neuronavigated cTBS to dmPFC and AI in a precise fashion.

The remaining three sessions were cTBS sessions (Sessions 3, 4, and 5; each 2.5-3 hours). Participants were told that their final payment would be computed based on their accuracy in the task. Every participant was paid £10/hour. In addition, all participants were paid up to £10 bonus in each session which was calculated based on their performance (£0 for 50% accuracy and £10 for 100% accuracy). Participants’ performance in the vertex session was used to calculate their bonus in all three sessions. Participants who completed all five cTBS sessions were given an extra £20 and a Persian chocolate at the end of the last session.

### Method details

#### Behavioral tasks

Experiments 1 and 2 used the same behavioral task, however, several changes were made to the task to adapt it to cTBS as discussed below.

##### Experiment 1

During the training part of the task, participants had to estimate seven characters’ scores in three trait domains: maths, memory, or planning. In each trial, participants were presented with an image of one of the seven characters. All faces were obtained from validated public face databases.^31^^,^^32^ The currently relevant trait was specified on top of the character’s image and was additionally indicated by the background colour (rose for math, arctic for memory, and lemon for planning). Participants indicated their estimate of the character’s score (see below on how different characters’ scores were generated) by using a mouse to move a blue dot along a horizontal line below the character’s image. After locking their estimate, veridical feedback about the character’s true task was indicated by a green dot on the horizontal line for three seconds. Each character was presented for two consecutive trials. Participants estimated the characters’ scores in each trait domain separately. Therefore, in each round of the training, the participants learned about seven characters’ abilities in one trait for two consecutive trials. After finishing each trait, they moved on to the next trait until all three traits were seen. They then started a second and a third round identical to the first round described above.

The range of the scores differed crosss the three traits. The scores ranged from zero to one (in maths), zero to ten (in memory) and zero to one hundred (in planning). Participants were told that the seven characters had participated in a different study and had completed three mini-tasks in which their abilities in maths, memory and planning were assessed. To familiarize the participants with how the scores were calculated, and to induce a profound context effect, the participants completed the three mini-tasks before starting the training (See Familiarization below). Unbeknownst to participants, the character scores were generated using a pseudorandom algorithm. To this end, we first created three (one for each trait) sets of scores (each with seven values, one for each of the seven characters) all between zero and one. The scores across different traits were similar in mean and variance. We used different sets of scores for the participants (6 sets in total). There was no other restriction on determining the scores. We multiplied the generated scores (which were originally between zero and 1) with 10 (for memory) and 100 (for planning). In the training part, on each trial, each character’s feedback was generated using a normal distribution centred on the score of that character and a very small standard deviation (σ =.02).

After the participants finished the training part, they started a test block in which they were presented with two characters sequentially and were instructed to choose the character with the higher score in the specified trait. The experiment consisted of four blocks, with 63 trials in each block. At the beginning of each trial, the relevant trait in which they had to compare the two characters was presented for 1 second. The screen’s background colour matched the background colour of the traits during the training. After that, the background colour turned grey, and a character was presented on the screen and disappeared after two seconds. The image of the first character was followed by the image of the second character which was presented for two seconds as well. The images of the two characters were separated by a jitter drawn from an exponential distribution with mean 4 s and truncated between 3-5 s. Participants were allowed to choose the character with the higher score as soon as the second character appeared on the screen. They could choose the first or the second character by pressing the left or right key on a button box, respectively. If the participants did not make a choice while the second character’s image was on the screen, they were given an additional three seconds to make a choice. If the participants failed to make a response within this time window, a text (“too slow”) cued them that the trial had been terminated. No trial-by-trial feedback was given during the test part. Instead, participants received cumulative feedback separately for each trait at the end of a test block. The cumulative feedback was computed as the proportion of correct choices for a given trait.

After the participants finished the block, they started a new training block. Like the previous training block, in each trial the participants were required to estimate the scores of the characters whose images were shown to them. However, this second training block differed from the first training block in some details. Most importantly, the trials were not separated by trait domain, but instead all traits and all characters were interleaved. After participants estimated all characters’ scores in all traits (each for one trial) and received feedback, they repeated the same procedure for another round. Therefore, overall, in the second training block, the participants estimated each character’s score in each trait twice. All other aspects of the second training block were identical to the first training block.

After the participants finished the second training block, they completed four test blocks in a 3T MRI scanner. All aspects of the test block were identical to the test block that they completed after the first training block outside the scanner. As mentioned above, in the test blocks, participants had to choose between all possible combinations of pairs of characters in all traits. On each trial, one of the characters was randomly selected to appear first and the other as the second character. However, if in block 1, a character appeared first in the comparison between character A and B in a specific trait (e.g., Maths), in the next block, character B appeared as the first character. With this arrangement, we guaranteed that, of the four comparisons that participants made between each pair of characters in each trait, both characters appeared as the first character twice.

*Familiarisation*: The aim of this part was to familiarise the participants with the tasks that they believed the characters completed to assess their scores in maths, memory, and planning.

*Maths*: The Maths task was the Number-Line Estimation task.^33^ Each trial involved a horizontal line with the left end labelled a number randomly drawn from a uniform distribution between 50 and 150. The right end of the line was labelled a number randomly drawn from a uniform distribution between 1300 and 1700. A yellow frame on top of the horizontal line indicated a number which was always within the range of the left and right end numbers of the line. After observing the target number, participants were required to move their cursor (a blue dot) using their mouse along the line and click the spot on the line that they estimated was closest to the target number. After participants confirmed their response by clicking on the line, a green dot showed the true location of the target number. In addition to this feedback, participants received a score about their performance. They were told that this score was calculated based on the precision of their estimate relative to the precision of the other participants who participated in the study in the past and that the characters’ score (which they were going to learn about in the training part) was calculated using the same procedure. In reality, to compute the score we first calculated participants’ errors by computing the distance between their estimate and the true location of the target number. This value was normalised by a factor guaranteeing that the error would lie within the zero to 1 range. From our pilot experiments we found that a normalisation factor of 200 would guarantee that most of the errors would lie within this range. We then subtracted the normalised error from 1 to create the final score. A mathematical summary of this procedure is presented below:

$$score=1-\left|{participant}^{\prime }s\;estimate-true\;location\right|/200$$

All scores below.1 or above.9 were replaced by a score sampled from a uniform distribution ranging from.1 to.25 or from.75 to.9, respectively. Participants completed five problems of this mini task.

*Memory*: In this mini-task, participants were required to remember the location of 9 blue dots on a 10 by 10 grid square and click on the dot locations after a short delay. To do that, participants were first presented with a 10 by 10 grid square for 2 seconds. Nine blue dots were randomly placed in any of the 100 cells in the grid square. Participants had 2 seconds to remember the location of the dots. After a short blank screen (1.5 seconds), participants were presented with the same grid square again and were asked to click on the cells in which they saw the dots. The grid remained on the screen until participants had selected nine cells. Each selected cell was indicated by a blue dot with that cell. After participants selected nine cells, they were given feedback about the number of cells they had correctly spotted. In addition to this, they also received a score between zero and 10. They were told that this score was computed relative to the performance of other participants that had previously participated in this test. To compute their scores, we multiplied the number of correct dots they remembered in each trial by a non-integer number (1.11) to make it more realistic to the participants that it was a relative score rather than simply the number of dots they correctly remembered. Participants completed 5 trials of this mini task. Participants were told that the characters’ memory scores in the main task was computed using the same procedure.

*Planning*: On each trial, participants were presented with the well-known Tower of London (ToL) task.^34^ The ToL consists of two boards. The top one is the target position and the bottom one is the starting configuration. Each board has three sticks, with several discs with different colours placed onto them. The participants’ task was to reconfigure the discs on the lower board (starting configuration) so that this board matched the target board. Between the two boards, there is a square referred to as the temporary position. To move a disc from one stick to another stick, participants had to first move it to the temporary position by clicking on the rectangle which covers the stick holding the disc. To move the disc from the temporary position to a stick, participants had to click on the stick onto which they wanted to move the disc. After completing each problem, participants received feedback about their performance. Participants were told that the feedback was computed based on i) the number of moves they took to solve the problem relative to the average number of moves that the previous participants took to solve the same problem, and ii) the time they spent solving it relative to the average time that the previous participants spent solving the problem. The average number of moves and the average time was computed using the pilot experiment performed prior to the main task. Participants completed five problems of this mini task, two problems with two discs, two problems with four discs and one problem with five discs.

##### Experiment 2

We modified several aspects of the experimental task to adapt it to the cTBS procedure for Experiment 2. The familiarisation and training parts were identical to the fMRI experiment (see above). Most importantly, in each session participants completed two test blocks in the test part, rather than four blocks as in the fMRI experiment. In addition, unlike the fMRI task procedure, participants did not receive cumulative feedback at the end of the test blocks. During the first experimental session, participants were familiarised with the three traits. Then, as in the fMRI procedure, they completed three rounds of training for each trait, followed by a test block and a further training block with the traits interleaved. They then completed two test blocks. The order of the test blocks in relation to cTBS application was counterbalanced across participants; stimulation was either applied immediately before the first test block, with a 40-minute break before the second block to allow for stimulation effects to diminish, or stimulation was applied 40 minutes after the first test block but immediately prior to the second test block. The order in which the traits were presented differed during each of the three experimental sessions.

*Regions of interest*: In Experiment 2, during each session we stimulated one of the three target stimulation sites; dorsomedial prefrontal cortex (dmPFC), anterior insula (AI), and vertex. We aimed to disrupt activity in the dmPFC and anterior insula while the vertex served as a non-active control condition. The three regions of interest were localised at the following MNI x/y/z-peak coordinates: dmPFC [2 60 28], AI [42 22 -8] or [46 18 0] (the former was preferable, but the latter was used for one participant as the coil was too close to the facial nerves), and vertex [0 -34 72]. The sites were then warped from the standard MNI space to individual structural images using FMRIB Software Library’s (FSL)^35^ non-linear transformations (FNIRT).

*cTBS protocol*: A MagStim-rapid-2 stimulator (MagStim, Whitland, Carmarthenshire, UK) connected to a 70mm figure-of-eight coil was used to deliver cTBS over scalp sites corresponding to the dmPFC, AI, and vertex. Transcranial magnetic stimulation was used on four occasions: during the first “taster” session to measure participants’ active motor threshold, and during the three experimental sessions in which neuronavigated cTBS was applied to each of the defined regions of interest. A standard cTBS protocol was followed during the experimental sessions.^36^ The three stimulation sites were projected onto individual high-resolution T_1_-weighted MRI brain scans using frameless stereotactic neuronavigation (Brainsight; Rogue Research). The nose, nasion, right ear and left ear were used for registration of the structural image. The cTBS intensity was set to 80% of the output of the cTBS machine at the participant’s active motor thresholds. This low threshold was chosen to limit the spread of stimulation from the target sites.^37^ 600 pulses in bursts of 3 pulses at 50Hz were applied every 200ms, leading to a total duration of approximately 40s. During stimulation, the coil was held in place tangentially to the skull by the researcher. The position of the cTBS coil with respect to the regions of interest was monitored online using the neuronavigation software.

Due to the proximity of the dmPFC and the AI to the facial nerves, the maximum allowed cTBS intensity was set to 50% of the machine’s maximum output. The one participant whose 80% active motor threshold was higher than this maximum level was excluded. Participants were instructed to limit their movements during the test block that immediately followed simulation.

### Quantification and statistical analysis

#### Behavioral data

##### LMM1 (Linear models for behavioral data analysis)

To investigate the effect of different task variables on choice in experiment 1, we conducted a linear mixed effect model as follow:

(Equation 1) $${choice}_{t}=\beta_{1s}+\beta_{2s}\times {rel_{value}}_{t}+\beta_{3s}\times {irel_{value}}_{t}+\beta_{4s}\times block+\beta_{5s}\times {\;rel_{value}}_{t}\times {irel_{value}}_{t}+\beta_{6s}\times {rel_{value}}_{t}\times block+\beta_{7s}\times {irel_{value}}_{t}\times block+\beta_{8s}\times {rel_{value}}_{t}\times {irel_{value}}_{t}\times block$$

Where ${choice}_{t}$ indicates participants’ choice on trial t and was set to 1 if character 1 was selected and 0 otherwise. ${rel_{value}}_{t}$ indicates the relevant trait domain of the first character minus the relevant trait domain of the second character on trial t. ${irel_{value}}_{t}$ indicates the irrelevant trait domain of the first character minus the irrelevant trait domain of the second character on trial t. The intercept ($\beta_{1s})$and all slopes ($\beta_{ks}$) were allowed to vary across participants by including random effects of the form $\beta_{ks}=\beta_{k0}+b_{ks}$ where $b_{ks}∼N\left(0,\sigma_{k}^{2}\right)$.

##### LMM2(Linear models for cTBS experiment)

For the analysis of experiment 2 (cTBS experiment), for each of the four trial types (congruent easy, incongruent easy, congruent hard, incongruent hard), we ran a linear logistic model in which we predicted choice using (a) the relevant and irrelevant value differences, (b) whether cTBS was applied before a block of trials, (c) block number, and (d) all possible interactions between these terms. We used the interaction between cTBS and the relevant value difference to quantify whether, or the way in which, cTBS changed the impact of relevant information on choice for a given combination of difficulty and congruency. Similarly, we used the interaction between cTBS and the irrelevant value difference to quantify whether, or the way in which, cTBS changed the impact of irrelevant information on choice. We initially included all regressors as mixed effects (like Equation 1), but the Hessian Matrix of the model was not positive definite, potentially due to overparameterization of the model (note that we had only two blocks in experiment 2 instead of four blocks like experiment 1). We therefore changed all the mixed effect variables in Equation 1 to fixed effect to ascertain a positive definite Hessian matrix which is indispensable for reliable beta estimation.

When we applied LMM1 after separating the trials based on congruency and difficulty, we orthogonalized the irrelevant traits with respect to the relevant trait to control for any collinearity induced by the separation of the data into these trial types.

In all linear regression models, we assessed the statistical significance of model parameters by F-statistics and Satterthwaite’s approximation for degrees of freedom.

#### fMRI data analysis

##### General

MRI data were analysed using Matlab (2020b) and Statistical Parametric Mapping software (SPM12; Wellcome Trust Centre for Neuroimaging, London, UK). Preprocessing comprised motion correction, correction for field inhomogeneity, spatial smoothing (5mm FWHM Gaussian kernel), realignment, coregistration, and normalisation to the MNI template and high-pass filtering (128 seconds) following SPM12 standard procedures.

##### Univariate analysis

The design matrix of GLM1 included seven regressors. Regressor 1-3: the context representation when the relevant trait domain was signalled (one regressor separately for each of the three contexts, 1 second boxcar). Regressor 4: first character presentation (2 seconds boxcar). Regressor 5: second character presentation (2 seconds boxcar). Regressor 6: for the extra choice time cue, which followed the second character presentation if no choice was made by that point (boxcar lasting the duration that the cue remained on the screen, maximum 3 seconds) Regressor 7: the time of button press (impulse function). We excluded trials where no response was made. Regressors 4 was parametrically modulated by relevant trait domain and the overall irrelevant trait domains. Regressor 5 was modulated by the relevant value (difference in the relevant trait domain between the chosen and unchosen characters) and irrelevant value (difference in the irrelevant trait domains between the chosen and unchosen characters).

##### Activity time course analysis

For each scan run, we regressed out variation due to head motion, and up-sampled the BOLD time course to a resolution of .2 seconds. For each trial, we extracted activity estimates in a 15 s window (75 time points), time-locked to 1 s before the onset of each event of interest. We used linear regression to predict the ROI activity time courses. More specifically, we applied a linear regression to each time point and then, by concatenating beta-weights across time points, created a beta-weight time course for each predictor of a regression model. We performed this step separately for each subject and pooled beta-weight time courses across subjects for visualisation. In the analysis of activity time courses aligned to the first character presentation, our predictors were the relevant and irrelevant trait values, the interaction between these terms and run number. In the analysis of the activity time course aligned to the second character presentation, our predictors were the relevant and irrelevant value differences, the interaction between these terms and run number. We tested group-level significance using a leave-one-out procedure to avoid any selection bias. For each participant and for each time course signal, we computed the peak signal (positive or negative) for the group and calculated the beta weight of the left-out participant at the time of the group peak. We repeated this procedure for each participant and compared the resulting beta weights against zero.

#### Multivariate analysis

##### Representational similarity analysis (RSA) in a priori regions of interests (ROI)

We used representational similarity analysis (RSA)^13^^,^^14^ to test whether the ROIs carried information about different task variables (i.e., relevant trait domains, context, characters’ identity) and also a 3D map of the social hierarchies. To estimate voxel activity patterns, we constructed an event-related GLM with the following regressors: a condition regressor locked to the context screen for each context (trait domain) introduced as a boxcar of 1 second in length, 21 character regressors (1 for each character in each trait domain) at the time of the first character presentation (averaged across all trials where each character was presented as the first character in each trait domain introduced as a boxcar of 2 seconds in length, one regressor for the presentation of the second character defined as a boxcar of 2 seconds in length, and finally one regressor for button press introduced as an impulse function. Regressors were convolved with a canonical hemodynamic response function. Regressors were modelled separately for each scan run, and constants were included to account for differences in mean activation between runs and scanner drifts.

We created a neural representational dissimilarity matrix (RDM) for each scan run. This RDM was constructed using the Mahalanobis distance (Euclidean distance after multivariate noise normalization) between the activity patterns for all possible pairings within our 3 (contexts) x 7 (characters) task design.^38^ Since the neural RDM was constructed using activity patterns from the same scan run (symmetric) and not different scan runs (cross-validated), we only used the upper triangle (and not the lower triangle and diagonal) of the resulting 21 x 21 neural RDM.

To test for the representation of the relevant trait domain, we created a model RDM where the distance between characters of the same rank in different contexts was set to zero and the distance between characters of different ranks was set to 1. Comparisons between characters within the same context was discarded to eliminate the effect of context (See Figure 3B). Testing for the representation of the characters’ identity was achieved by creating a model RDM where the same characters across different contexts was set to zero and distance between different characters across contexts was set to 1. Again, comparisons between characters within the same context was discarded to eliminate the effect of context. To compute the model RDM for testing the representation of overall rank, we first computed the characters overall rank across all three domains. Each cell of the model RDM then represented the distance of the overall rank between each pair of characters. Test for the representation of the context was done by creating a model RDM where the cells in the same contexts were set to zero while all other cells were set to 1. We used rank correlation (Kendall’s $\tau_{A}$) to quantify the extent to which pattern activity within each ROI was explained by a model RDM. We used rank correlation (Kendall’s $\tau_{A}$) to quantify the extent to which a neural RDM was explained by a model RDM. Specifically, we first computed the correlation between the model and neural RDMs for each scan run and then averaging the correlations across the four runs for subsequent group analysis. As described above, we only used the upper triangle of the RDMs for analysis. The HC ROI was defined using a previous study.^39^ The EC ROI was defined by Juelich atlas.^40^

##### Whole brain RSA

Whole-brain searchlight RSA was performed to examine brain areas in which the activity patterns carried information about participants’ overall impression of the characters defined as the average rank across all three trait domains and also a 3D representation of trait domains defined as the Euclidian distance between characters across all three trait domains. We defined a sphere containing 100 voxels around each searchlight centre voxel. The dissimilarity matrices were quantified with the Euclidean distance between neural patterns estimated from different blocks. The model RDM for overall rank and 3D distance were created using the procedure explained above. Again, we used rank correlation (Kendall’s $\tau_{A}$) to quantify the extent to which pattern activity within each searchlight was explained by the model RDM.

##### Correction for multiple comparison

For whole brain analyses, in both univariate and multivariate analyses, group level testing was done using a one-sample t-test on the functional maps generated by the first level analysis. We used the maximum cluster mass statistic^41^ for multiple comparisons correction. We reported clusters that survived FWE correction at a cluster-forming threshold of p < 0.001. For other behavioral and time course analyses, we used Holm-Bonferroni method^42^ to account for multiple comparison problem, where applicable.

##### Anatomical location specification

We used the Harvard-Oxford probabilistic atlas (https://neuro.debian.net/pkgs/fsl-harvard-oxford-atlases.html) to locate the relevant cluster in Figure 3C. The atlas calculated that there was a 70% probability that this cluster was within AI.

## Data Availability

The data and code that support the findings of this study are available via Figshare (https://doi.org/10.6084/m9.figshare.21510831.v1). Any additional information required to reanalyse the data reported in this paper is available from the lead contact upon request.

## Supplemental information

Document S1. Figures S1–S8, Tables S1–S4, and supplemental references

Document S2. Article plus supplemental information
