# Supplementary material for: Causal role of a neural system for separating and selecting multidimensional social cognitive information
Source: Neuron. 2023 Apr 5;111(7):1152–1164.e6. doi: 10.1016/j.neuron.2022.12.030 (PMC10914676; doi:10.1016/j.neuron.2022.12.030)
Supplement: Document S1. Figures S1–S8, Tables S1–S4, and supplemental references [file mmc1.pdf]

Neuron, Volume *111*

Supplemental information

Causal role of a neural system for separating and selecting multidimensional social cognitive information

Ali Mahmoodi, Hamed Nili, Caroline Harbison, Sorcha Hamilton, Nadescha Trudel, Dan Bang, and Matthew F.S. Rushworth

Supplemental Information

Causal role of a neural system for separating and selecting multidimensional social cognitive information

Primary Supplemental PDF:

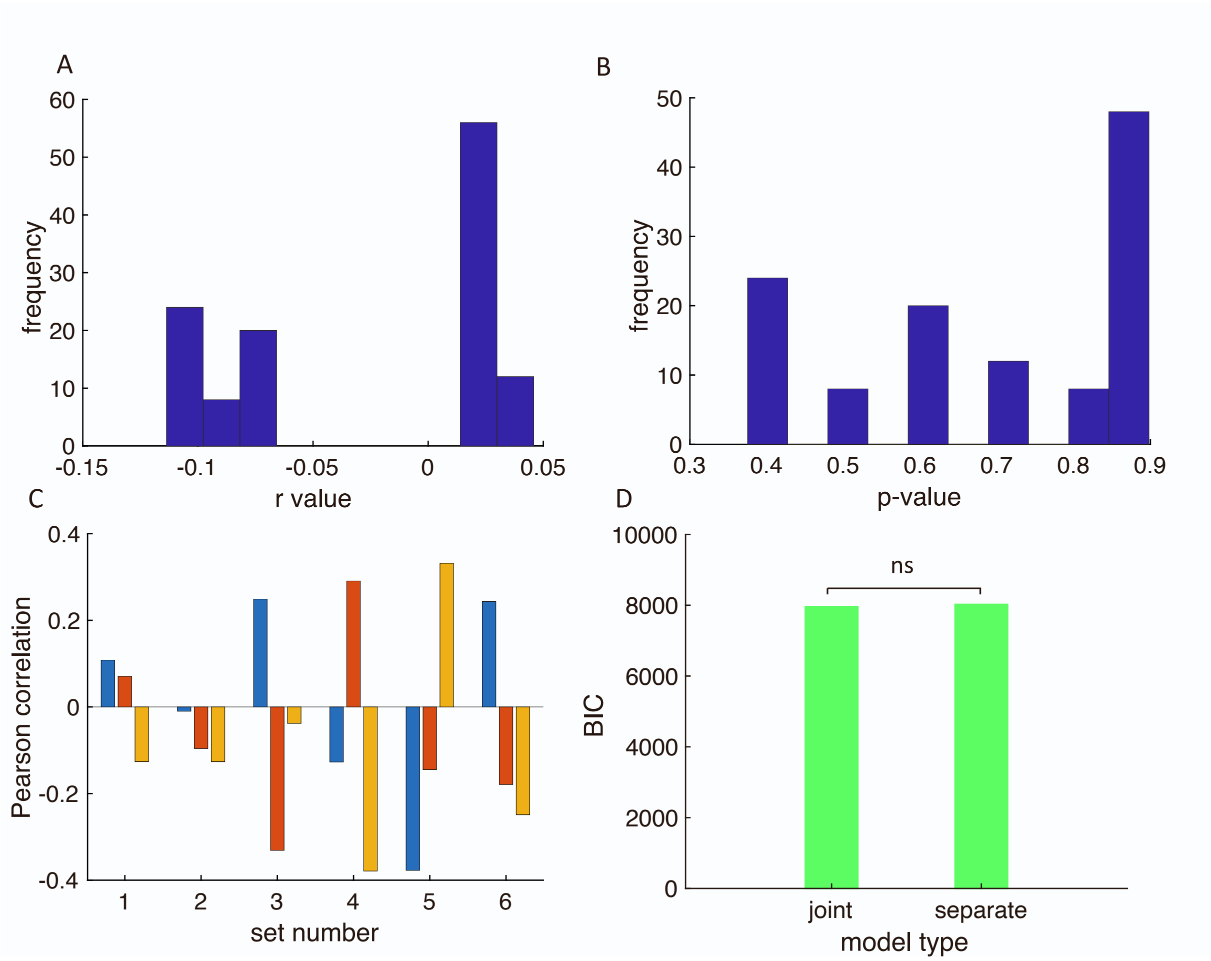

Figure S1. Task design and model comparison, related to Figure 1 and 2. A We computed the correlation between relevant and irrelevant trait domains separately for each block and each character as they were put in the LMM1. The y-axis indicates the frequency of the Pearson r. **B** similar to **A** but the frequency is plotted against p-values obtained from Pearson r in A. **C** Pearson correlation (y-axis) was computed for each pair of the three trait domains. Each colour indicates each pairwise correlation between the three trait domains (three pairs for each set). **D** We tested an alternative model to LMM1 in the main text where, instead of averaging the two irrelevant trait dimensions into a single irrelevant value difference (joint model), we separated them into a high and a low irrelevant value difference (separate model). In support of using a single irrelevant value difference, both terms had a positive effect on choice (high $\beta \pm 95\%$ CI = .04 ± .02, t(7544) = 4, p < .001, HBC; low $\beta \pm 95\%$ CI = .04 ± .02, t(7544) = 3.7, p < .001, HBC). Even though this alternative model provided a better fit than the null model without irrelevant information (Likelihood-Ratio test, p < .001), it did not provide a better fit than the original model which included a single irrelevant value difference (Likelihood-Ratio test, p = .35). In addition, we highlight that our logistic mixed effects model provided a better fit than a null model without irrelevant traits (Likelihood-Ratio test, $\chi^2(11)$ = 185.91, p < .001, HBC). We obtained the same results when we separated the trials into four categories based on congruency and difficulty (Likelihood-Ratio test; incongruent easy, $\chi^2(9)$ = 10, p =.03; congruent easy, $\chi^2(9)$ = 19, p =<.001; incongruent hard, $\chi^2(9)$ = 12, p =.01; congruent hard, $\chi^2(9)$ = 12, p =.01).

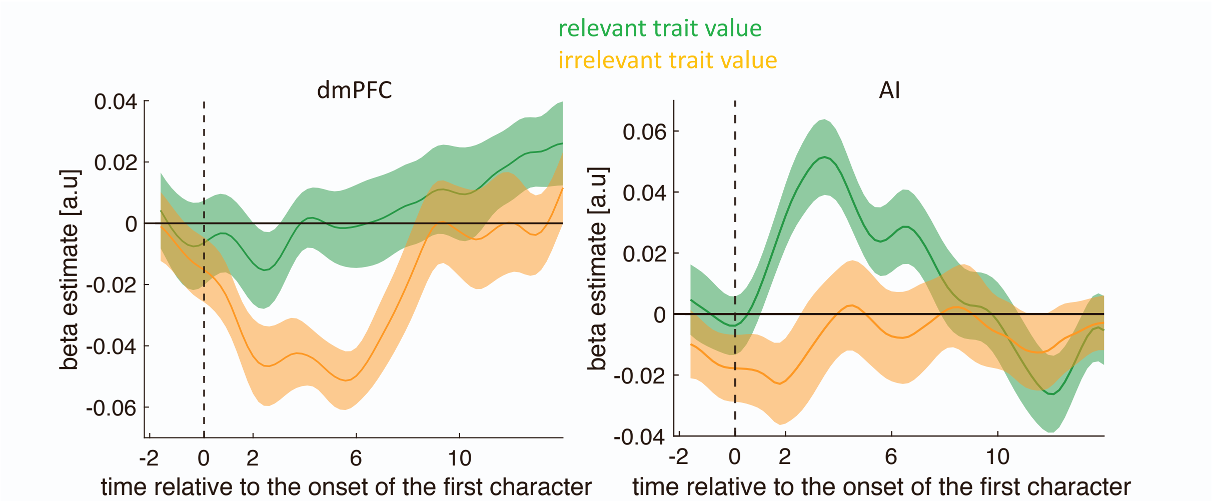

Figure S2. AI and dmPFC activity at first character presentation, related to Figure 3. At the time of the first character, while the dmPFC encoded the irrelevant trait domain (consistent with Figure 3 in the main text) but not the relevant (left panel, Wilcoxon signed-rank test, relevant W = 200, p = .25, irrelevant W = 44, p <.001), AI encoded the relevant trait domain but not the irrelevant trait domain (right panel, Wilcoxon signed-rank test, relevant W = 409, p = <.001, irrelevant W = 64, p = .99).

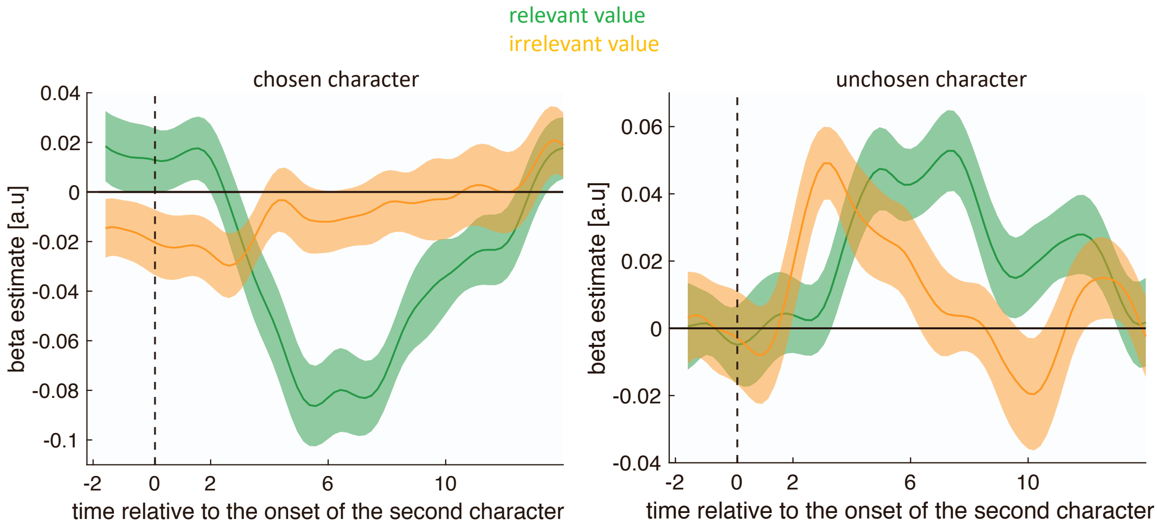

Figure S3. Unpacking AI response to value difference at second character presentation, related to Figure 3. There are different possible explanations of the negative relationship between AI activity and the difference between the chosen and unchosen in the relevant trait domain (relevant value difference). First, AI activity may be negatively correlated with the value of the chosen character (BOLD decreases as the value of the chosen character increases). Second, AI activity may be positively correlated with the value of the unchosen character (BOLD increases as the value of the unchosen character increases). A third possibility is a combination of the two former accounts (BOLD increases as relevant value difference decreases). To tease apart these possibilities, we applied two separate regression analyses to the AI activity time course. In the first "chosen-character" analysis, we predicted AI activity using the relevant value of the chosen character, the irrelevant value of the chosen character and their interaction. In the second "unchosen-character" analysis, we predicted AI activity using the relevant value of the unchosen character, the irrelevant value of the unchosen character and their interaction. In line with the third possibility and consistent with the view that this signal encoded a comparison process, these analyses indicated that AI encodes the relevant value of the chosen character with a negative sign and the relevant value of the unchosen character with a positive sign (Figure S3, Wilcoxon signed-rank test: chosen character, relevant $W = 8$ $p <.001$, irrelevant $W = 132$, $p =.01$, unchosen character, relevant $W = 366$ $p =.003$, irrelevant $W = 385$, $p <.001$). As a result, AI activity is more negative when the decision is easy (high value difference) than when the decision is difficult (low value difference). Notably, multiple studies suggest that both negative and positive encoding of the difference in value between a chosen and an unchosen option indicate a comparison process. While the sign of this relationship is typically positive in human ventromedial prefrontal cortex (BOLD increases as the value difference increases), it tends to be negative in macaque ventromedial prefrontal cortex (see[1] for more details). Critically, network modelling [2] indicates that both response profiles are consistent with a comparison process but arise due to different network configurations (see Figure S9 in [2]). In other brain areas such as anterior cingulate cortex and the intraparietal sulcus, decision-related activity is negatively related to the difference in value between a chosen and an unchosen option in both species [3,4]. Thus, the negative relationship found between the difference in value between a chosen and an unchosen option is similar in kind to that seen in several other brain areas that have been linked to decision making.

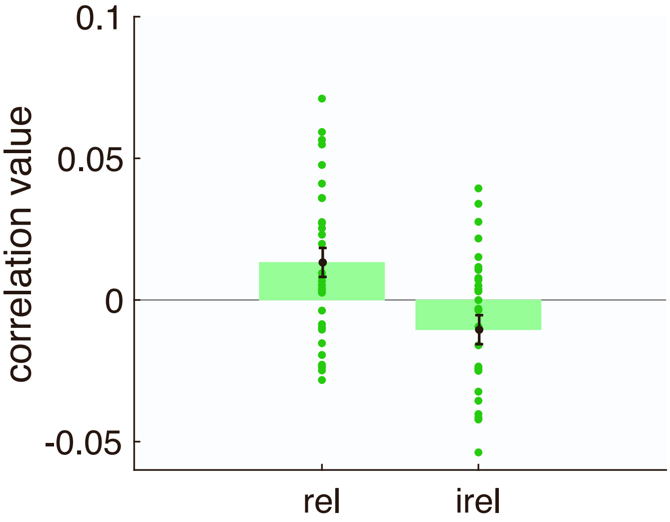

Figure S4. Encoding of ordered relationship between ranks in dmPFC, related to Figure 4. In the main text, we show that multivariate dmPFC activity distinguishes between different ranks. However, dmPFC may not only carried such a categorical rank representation but also be sensitive to the ordered relationships between ranks. To address this question, we created model RDMs based on the rank distance between the characters in the relevant and irrelevant trait domains. Intriguingly, multivariate dmPFC activity carried not only a categorical but also a parametric rank representation in the relevant domain (Wilcoxon-signed rank test $W = 336$, $p = .01$). In line with results reported in the main text, we found no evidence for a parametric rank representation in the irrelevant domains (Wilcoxon-signed rank test $W = 154$, $p = .94$).

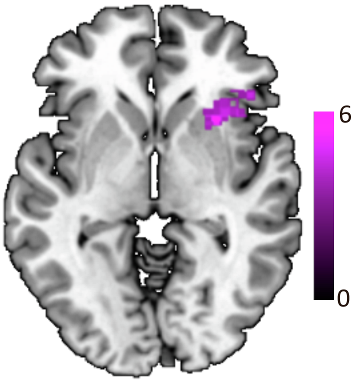

Figure S5. Encoding of overall rank in AI at first character presentation, related to Figure 4. using representational similarity analysis (RSA), we found that a cluster in the right AI carried information about characters’ overall rank at the time of first character presentation.

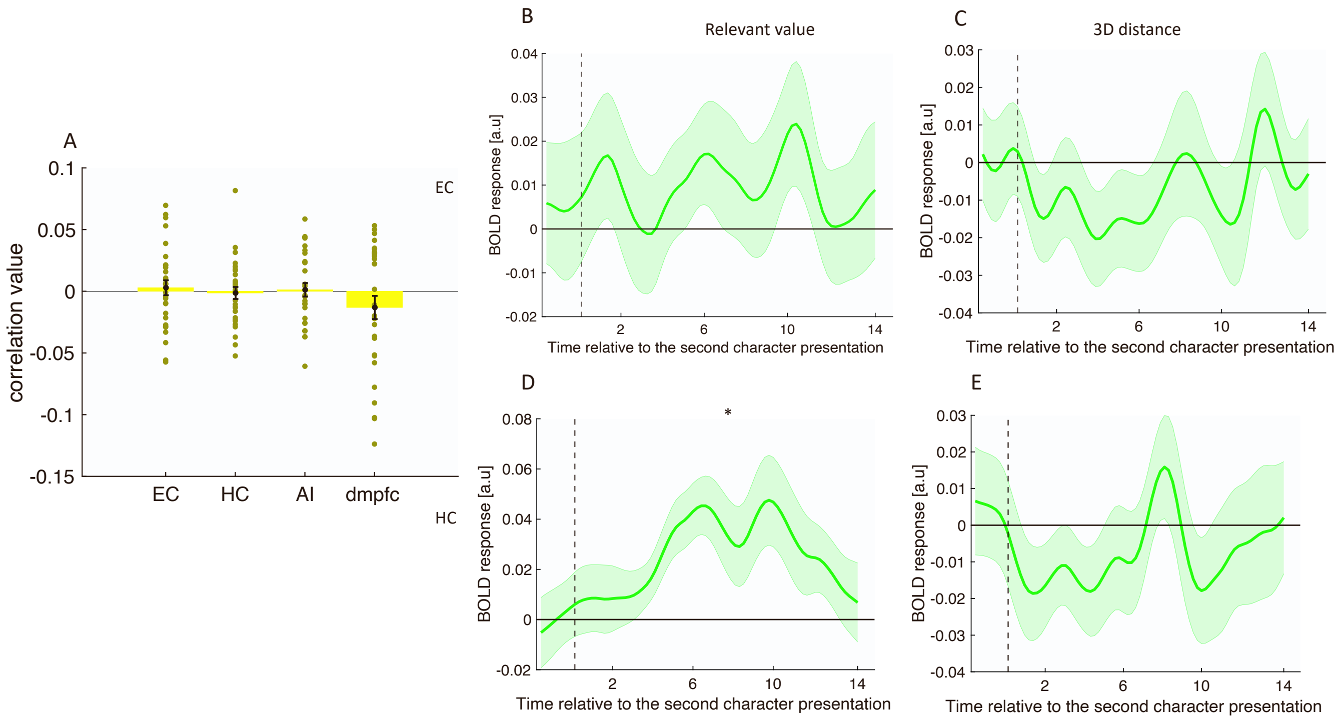

Figure S6. Investigating 3D rank representation and 3D value difference related to Figure 3 and Figure 4. A Correlation value between brain activity pattern and behavioural RDM defined as 3D Euclidian distance for four brain areas (EC, HC, AI, and dmPFC). One concern is whether null results reflect a genuine lack of an effect or a lack of statistical power. To address this issue, we computed Bayes factors for the four ROIs where $1/10 < BF_{10} < 1/3$ indicates moderate evidence for the null hypothesis (evidence of absence), whereas BF_{10} values between 1/3 and 3 indicate that there is insufficient evidence to draw a conclusion (absence of evidence) [5–7]. In all four ROIs, there was moderate evidence for the null hypothesis (EC BF_{10} = .23, HC BF_{10}= .15, AI BF_{10}= .15, dmPFC BF_{10} = .09), indicating that our task did not involve a 3D social hierarchy representation. **B-E** Correlates of relevant value (defined as the difference in relevant trait between the chosen and unchosen characters) (B and D) and 3D distance (the Euclidian distance between the two characters across all three domains) (C and E) in the EC-HC system at the time of choice. We found that only HC encoded the difference between the chosen and unchosen characters (D), while neither EC nor HC encoded Euclidian distance between characters across all three domains (C and E).

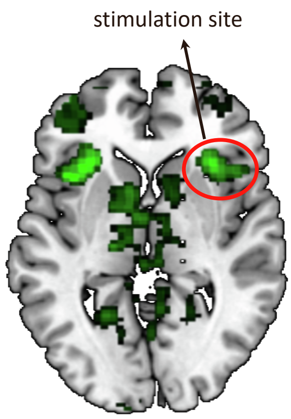

Figure S7: activity of anterior insula at the time of choice at a lower threshold (p = .05, uncorrected), related to Figure 3 and Figure 6.

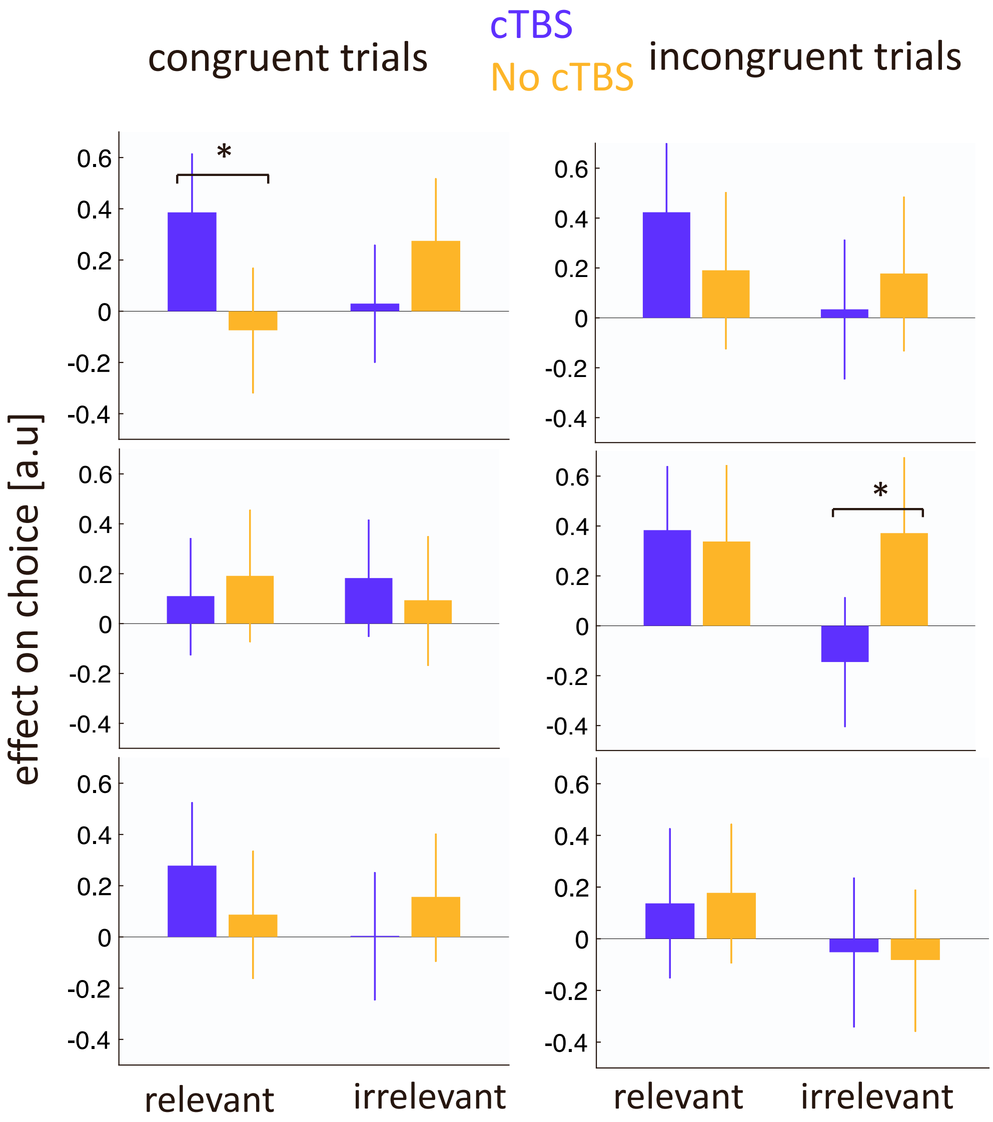

Figure S8. Detailed cTBS results, related to Figure 6. In the main text, we reported on regression analyses in which we included both experimental blocks (“cTBS” and “No cTBS”) in each analysis (Figure 6). Here, we run the regression analyses for each of the four combinations of congruency and difficulty separately for the “cTBS” and “No cTBS” blocks. This figure indicates the effect of relevant and irrelevant information on choice in “cTBS” and “No cTBS” for hard trials only. The statistics for both hard and easy trials are reported in Tables S4.

Trait	Cluster size	T	Z	Peak MNI coordinates		
				x	y	z
Relevant	638	5.94	4.77	-44	-36	46
	72	4.49	3.88	24	-56	-20
Irrelevant	229	5.85	4.72	-42	-40	40
	91	4.62	3.97	-32	-86	-8

Table S1. Brain areas that significantly correlated with each trait dimension at the time of second character presentation, related to Figure 3.

Correlation	Cluster size	T	Z	Peak MNI coordinates		
				x	y	z
Negative	526	6.54	5.09	-50	22	32
	299	6.33	4.98	-34	22	-2
	949	5.93	4.76	0	14	52
	173	5.23	4.36	-28	-54	44
	260	5.18	4.32	38	22	-6
	114	4.65	3.99	50	24	32
Positive	138	5.13	4.29	-58	-32	32
	200	5.07	4.25	62	-30	30

Table S2. Brain areas that were correlated with relevant value at the time of second character presentation, related to Figure 3.

ROI	Condition	Coefficient/congruency	Congruent Beta±CI, F Statistic, p-value	Incongruent Beta±CI, F Statistic, p-value
AI	Easy	relevant	.27±.38, F(1,284) = 1.9, p=.16	-.12±.36, F(1,340) =.44, p=.5
		irrelevant	-.25±.38, F(1,284) = 1.65, p=.19	.2±.35, F(1,340) =1.21, p=.27
dmPFC	Easy	relevant	.01±.32, F(1,277) = .004, p=.94	-.06±.36, F(1,334) =.12, p=.72
		irrelevant	-.15±.32, F(1,277) = .97, p=.32	.33±.35, F(1,334) =3.31, p=.06
vertex	hard	relevant	.19±.35, F(1,636) = 1.11, p=.29	-.04±.39, F(1,504) =.04, p=.83
		irrelevant	-.15±.35, F(1,636) = .7, p=.4	.03±.39, F(1,504) =.02, p=.88
	Easy	relevant	-.12±.32, F(1,284) = .57, p=.44	.19±.34, F(1,340) =1.2, p=.27
		irrelevant	.18±.3, F(1,284) = 1.41, p=.23	.22±.34, F(1,340) =1.6, p=.2

Table S3. Impact of the cTBS on the effect of the relevant and irrleavnt information on choice, related to Figure 6.

ROI	Condition	Coefficient		Congruency	
				Congruent<br>Beta±CI, F Statistic, p-value	Incongruent<br>Beta±CI, F Statistic, p-value
dmPFC	Easy	Relevant	TMS	.19 ± .27, F(1, 311) = 2.01, p = .16	.34 ± .31, F(1, 248) = 4.51, p = .03
			No-TMS	.11 ± .24, F(1, 311) = .83, p = .36	.38 ± .26, F(1, 247) = 8.23, p = .004
		Irrelevant	TMS	.09 ± .26, F(1, 311) = .49, p = .48	.37 ± .31, F(1, 248) = 5.72, p = .02
			No-TMS	.18 ± .24,F(1, 311) = 2.32, p = .13	-.15 ± .26, F(1, 247) = 1.21, p = .27
	Hard	Relevant	TMS	.29 ± .23, F(1, 138) = 6.15, p = .01	.37 ± .28, F(1, 167) = 7.0, p=.01
			No-TMS	.27 ±.22, F(1, 139) = 5.81, p = .02	.42 ± .24, F(1, 167) = 12.5, p = .001
		Irrelevant	TMS	.02 ±.23, F(1, 138) = .02, p = .87	.18 ± .28, F(1, 167) = 1.64, p = .20
			No-TMS	.17 ± .22, F(1, 139) = 2.31, p = .13	-.15 ± .23, F(1, 167) = 1.52, p = .22
AI	Easy	Relevant	TMS	.51 ± .26, F(1, 142) = 14.9, p <.001	.38 ± .28, F(1, 170) = 7.38, p = .01
			No-TMS	.24 ± .29, F(1, 142) = 2.70, p = .10	.51 ± .23, F(1, 170) = 18.5, p < .001
		Irrelevant	TMS	-.13 ± .27, F(1, 142) = .93, p = .34	.21 ± .28, F(1, 170) = 2.33, p = .13
			No-TMS	.13 ± .29, F(1, 142) = .76, p = .38	.01 ± .23, F(1, 170) = .01, p = .91
	Hard	Relevant	TMS	-.07 ± .25, F(1, 318) = .35, p = .55	.19 ± .32, F(1, 252) = 1.41, p = .24
			No-TMS	.39 ± .23, F(1, 318) = 10.88, p = .001	.42 ± .28, F(1, 252) = 8.80, p = .003
		Irrelevant	TMS	.27 ± .25, F(1, 318) = 4.82, p = .03	.18 ± .31, F(1, 252) = 1.26, p = .26
			No-TMS	.03 ± .23, F(1, 318) = .06, p = .80	.03 ± .28, F(1, 252) = .06, p = .81
Vertex	Easy	Relevant	TMS	.49 ± .25, F(1, 142) = 15.4, p <.001	.39 ± .24, F(1, 170) = 10.1, p = .002
			No-TMS	.37 ± .21, F(1, 142) = 11.7, p = .001	.58 ± .24, F(1, 170) = 22.8, p <.001
		Irrelevant	TMS	-.13 ± .24, F(1, 142) = 1.11, p = .29	.01 ± .24, F(1, 170) = .01, p = .92
			No-TMS	.06 ± .21, F(1, 142) = .34, p = .56	.24 ± .25, F(1, 170) = 3.59, p = .06
	Hard	Relevant	TMS	.09 ± .25, F(1, 318) = .47, p = .50	.18 ± .27, F(1, 252) = 1.64, p = .20
			No-TMS	.28 ± .25, F(1, 318) = 4.70, p = .03	.14 ± .29, F(1, 252) = .87, p = .35
		Irrelevant	TMS	.16 ± .25, F(1, 318) = 1.48, p = .22	-.08 ± .28, F(1, 252) = .34, p = .56
			No-TMS	.004 ± .25, F(1, 318) = .001, p = .97	-.05 ± .29, F(1, 252) = .12, p = .72

Table S4: Regression analyses results for each of the four combinations of congruency and difficulty separately for the “cTBS” and “No cTBS” blocks, related to Figure 6.

Supplementary references:

1. Klein-Flügge, M.C., Bongioanni, A., and Rushworth, M.F. (2022). Medial and orbital frontal cortex in decision-making and flexible behavior. Neuron.

2. Papageorgiou, G.K., Sallet, J., Wittmann, M.K., Chau, B.K., Schüffelgen, U., Buckley, M.J., and Rushworth, M.F. (2017). Inverted activity patterns in ventromedial prefrontal cortex during value-guided decision-making in a less-is-more task. Nat. Commun. *8*, 1–14.

3. Hunt, L.T., Kolling, N., Soltani, A., Woolrich, M.W., Rushworth, M.F., and Behrens, T.E. (2012). Mechanisms underlying cortical activity during value-guided choice. Nat. Neurosci. *15*, 470.

4. Rushworth, M.F., Kolling, N., Sallet, J., and Mars, R.B. (2012). Valuation and decision-making in frontal cortex: one or many serial or parallel systems? Curr. Opin. Neurobiol. *22*, 946–955.

5. Jeffreys, H. (1998). The theory of probability (OUP Oxford).

6. Keysers, C., Gazzola, V., and Wagenmakers, E.-J. (2020). Using Bayes factor hypothesis testing in neuroscience to establish evidence of absence. Nat. Neurosci. *23*, 788–799.

7. Lee, M.D., and Wagenmakers, E.-J. (2014). Bayesian cognitive modeling: A practical course (Cambridge university press).
